# Rectovaginal fistulas after radiation therapy: treatment options and outcomes – a narrative review

**DOI:** 10.3389/fonc.2026.1928527

**Published:** 2026-09-07

**Authors:** Katarzyna Lachowska, Nina Jankowska, Anna Januszek, Julia Orzelska, Natalia Gierulska, Adrianna Kwiatkowska, Piotr Kuchno, Dominika Ozimek, Krzysztof Kułak, Rafał Tarkowski

**Affiliations:** 1 Student’s Scientific Association at the I Chair and Department of Gynaecological Oncology and Gynaecology, Medical University of Lublin, Lublin, Poland; 2 I Chair and Department of Gynaecological Oncology and Gynaecology, Medical University of Lublin, Lublin, Poland

**Keywords:** coloanal anastomosis, graciloplasty, Martius flap, pelvic reconstruction, radiation therapy, rectovaginal fistula, regenerative medicine, stromal vascular fraction

## Abstract

**Introduction:**

Radiation-induced rectovaginal fistula (RI-RVF) is a severe late complication of pelvic radiotherapy, arising predominantly after treatment for gynaecological malignancy and characterised by fibrosis, ischaemia, impaired healing and poor quality of life. No universally accepted treatment standard exists.

**Methods:**

A structured narrative review searched PubMed and Google Scholar (January 2020–July 2026), with earlier landmark studies identified through reference-list screening; it was not conducted or reported as a systematic review. Eight reviewers screened records singly rather than in duplicate, with uncertain cases resolved by consensus with two senior supervisors. Predefined outcome domains beyond anatomical closure were extracted, including stoma-free survival, continence, low anterior resection syndrome, sexual function, pelvic pain and patient-reported quality of life. Seventy publications were cited, of which 26 constitute the clinical evidence base; the remaining 44 provided anatomical, radiotherapeutic, methodological or background context.

**Results:**

Faecal diversion is an appropriate initial, bridging or palliative intervention rather than a universal first-line treatment; spontaneous closure after diversion alone is uncommon (6/50; 12%), and colostomy and ileostomy carry distinct complication profiles. The Martius flap is applied mainly to low fistulas with small defects; the quoted 1.5 cm threshold reflects selection criteria in published series rather than a validated cut-off, and larger defects have been repaired successfully. Gracilis interposition is used for complex or recurrent fistulas, although supporting meta-analytic data derive from heterogeneous perineal fistula cohorts in which only approximately 18% of patients had received radiotherapy. Delayed coloanal anastomosis was associated with fewer anastomotic complications than immediate anastomosis in low rectal cancer meta-analyses, though not consistently across endpoints; this evidence does not derive from RI-RVF populations. Functional and patient-reported outcomes were reported inconsistently, with non-comparable instruments. Evidence for stromal vascular fraction is confined to case reports and small series.

**Conclusions:**

The overall certainty of the available evidence is low. Martius flap reconstruction, graciloplasty and delayed coloanal anastomosis are established options, but reported efficacy must be interpreted against the heterogeneity and indirectness of the available evidence. Stromal vascular fraction remains experimental. Prospective studies should redefine success beyond anatomical closure to include stoma-free survival, functional recovery and patient-reported quality of life.

## 1 Introduction

Rectovaginal fistula (RVF) is an abnormal connection between the rectum and vagina (1). It is one of the more serious late stage complications resulting from radiotherapy administered for malignancies of the pelvic region (2, 3). Patients with this condition often have a history of undergoing radiotherapy several years earlier, typically for gynecological cancers such as cervical or endometrial cancer (4, 5). Reported incidence varies with the population studied, the era of treatment and the interval since radiotherapy, and figures between approximately 3% and 13% have been cited for women treated for cervical cancer (3, 4). It is a debilitating anorectal disorder that markedly impairs patients’ quality of life. Besides radiotherapy, RVFs may also occur as a consequence of obstetric injury, inflammatory bowel disease, such as Crohn’s disease, or as postoperative complications following pelvic surgery (1, 2, 6, 7).

The presence of an RVF results in profound physical and psychological burden, with the most distressing clinical manifestation being the passage of fecal matter, air or even purulent discharge through the vaginal canal (2, 7). Furthermore, the persistent and intractable nature of these symptoms frequently precipitates psychological morbidity, often leads to significant sexual dysfunction, such as dyspareunia and severely restricts social engagement (2, 7, 8). Inaddition, patients suffer from radiation proctitis, which is a form of refractory anorectal symptoms. These include rectal bleeding, perianal pain and tenesmus (2, 6). RVFs also cause vaginal irritation, perianal pain, and recurrent genitourethral infection (7).

Diagnosing RVF fundamentally depends on patient presentation and clinical examination, alongside an optional methylene blue test. To achieve a comprehensive and objective assessment, ultrasound and magnetic resonance imaging (MRI) are typically performed (9). Radiation injury to the rectovaginal septum affects all layers of the bowel and vaginal wall. Progressive obliterative endarteritis of small arterioles leads to hypoperfusion and chronic ischaemia, and the irradiated submucosa and muscularis are gradually replaced by dense, inelastic fibrotic tissue (3).

A definitive standard treatment option for the condition is lacking, despite the many surgical options that are available, therefore decisions must be made on an individual basis (7).

This article aims to discuss the methods available for treating RVFs resulting from radiotherapy, and to evaluate their effectiveness based on the available literature.

## 2 Methods: literature search and selection

This article is a structured narrative review with a documented search. It was not conducted or reported as a systematic review: no protocol was registered, PRISMA reporting was not applied, records were not screened in duplicate, and no formal risk-of-bias instrument was used. The narrative label is retained deliberately, because the heterogeneity of study designs, populations and outcome definitions precluded quantitative synthesis. The search and selection process is reported in as much detail as the retrospective documentation permits. The article should therefore be read as a narrative review with a structured search rather than as a systematic or fully reproducible evidence synthesis.

### 2.1 Search strategy and sources

The primary literature search was performed in PubMed and was last updated on 31 July 2026. Google Scholar was searched additionally to identify records not indexed in PubMed; this supplementary search was not conducted systematically and its yield was not recorded.

The primary database search was conducted for literature published between January 1, 2020, and July 31, 2026. The search strategy combined MeSH and text words. Each term listed below was searched in combination with “rectovaginal fistula” as a separate query rather than as a single Boolean string; the individual result sets were then merged, which yielded 5,642 records in total. The terms were: “radiotherapy”, “radiation-induced rectovaginal fistula”, “diagnosis”, “treatment”, “gracilis flap”, “graciloplasty”, “Martius flap”, “coloanal anastomosis”, “colostomy”, and “stromal vascular fraction”.

### 2.2 Criteria for inclusion and selection of publications

The primary inclusion criteria were (1): studies focusing on the diagnosis, surgical repair, or regenerative management of rectovaginal fistulas; (2) studies specifically reporting outcomes in patients with radiation-induced fistulas or complex pelvic fistulas; (3) peer-reviewed original clinical research, systematic reviews, meta-analyses, or clinical case series; and (4) articles published in English. While primary literature selection focused on recent evidence (2020–2026), pioneering landmark studies and key historical clinical series published prior to 2020 were manually identified through reference list screening and included to provide necessary procedural baselines and long-term historical context.

Screening was distributed among eight reviewers (K.L., N.J., A.J., J.O., N.G., A.K., P.K., D.O.). Each record was assessed by one reviewer working independently; records were not screened in duplicate. Records whose eligibility was uncertain at title, abstract or full-text stage were referred for discussion and resolved by consensus with two senior supervisors (K.K., R.T.).

Articles were excluded if they met any of the following criteria: duplicates, studies lacking methodological clarity or adequate clinical data, and abstract-only conference proceedings. Methodological quality of included studies was appraised narratively based on sample characterization, clarity of surgical technique, and reporting of follow-up outcomes; no formal risk-of-bias instrument was applied. Data extraction was performed using a standardized grid capturing study design, patient characteristics, intervention type, fecal diversion practices, closure rates, follow-up durations, and adverse events. In addition to anatomical closure, predefined outcome domains were extracted wherever reported: stoma-free survival, stoma reversal, faecal continence, low anterior resection syndrome, vaginal stenosis, sexual function and dyspareunia, pelvic pain, reoperation burden, and patient-reported quality of life. Domains not reported in a given study were recorded as such, and this was retained as a finding. Throughout this review, “closure” denotes absence of the fistula tract on clinical or radiological assessment; “primary healing” denotes closure after the index procedure without further intervention; and “final healing” denotes closure achieved after any number of additional procedures. Definitions of success and minimum follow-up periods varied across the included studies and are reported individually in **Supplementary Table 1**.

**Figure 1:**
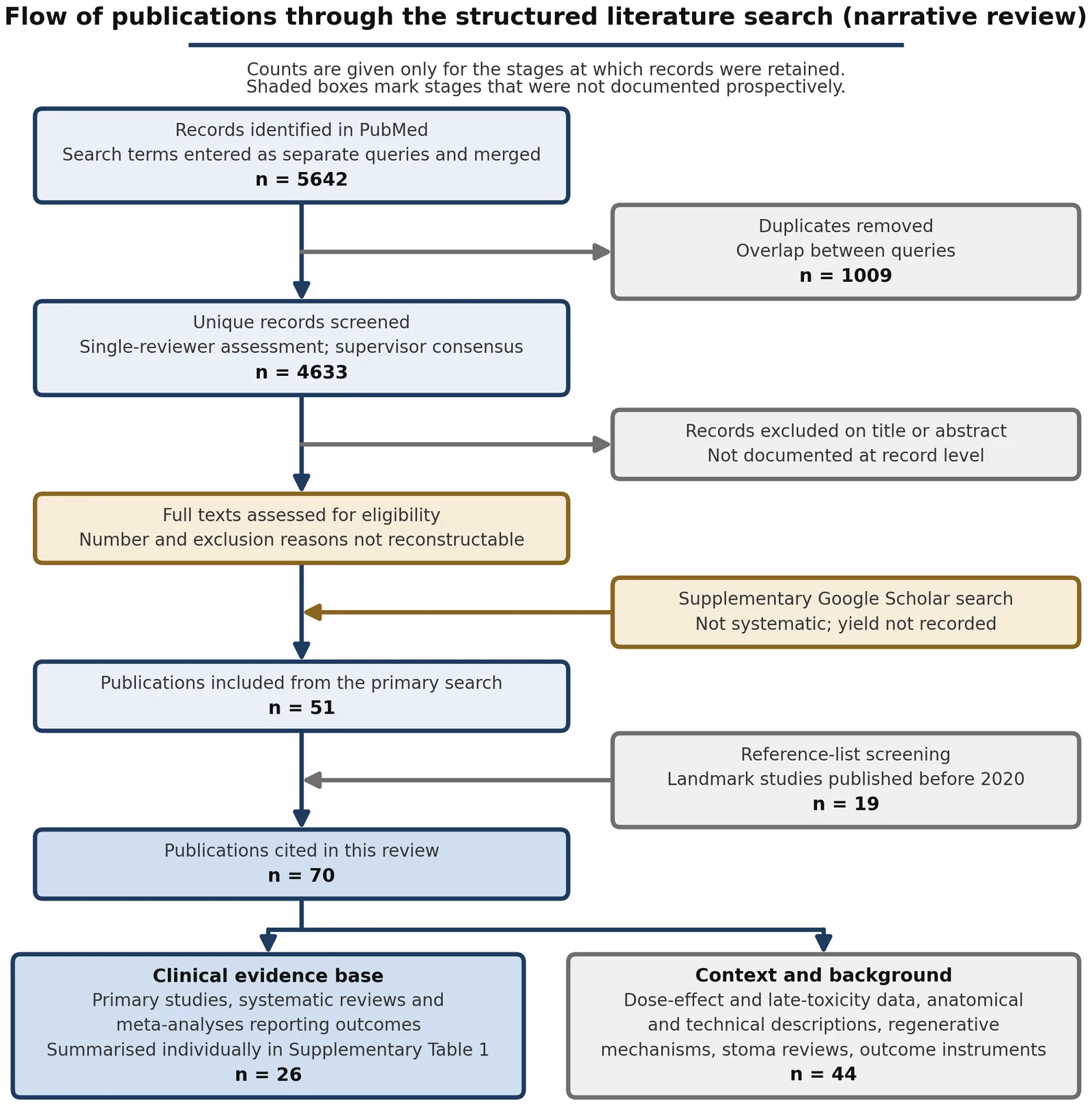
Flow of publications through the structured literature search performed for this narrative review. This is a descriptive diagram of the process followed and is not a PRISMA flow diagram; no protocol was registered and PRISMA reporting was not applied. Counts are given only for the stages for which records were retained. Search period 1 January 2020 to 31 July 2026; the primary search was performed in PubMed and last updated on 31 July 2026. Search terms were entered as separate PubMed queries and the result sets merged, so that the duplicates removed represent overlap between queries rather than duplicate indexing. Each record was assessed by a single reviewer, with uncertain cases resolved by consensus with two senior supervisors; screening was not documented at the level of individual records, and the number of full texts assessed and the reasons for exclusion at that stage could not be reconstructed. Google Scholar was searched to identify records not indexed in PubMed, but this supplementary search was not systematic and its yield was not recorded. Eligibility was restricted to English-language publications.

Because several terms retrieved overlapping sets, 1,009 records were duplicated across queries and were removed, leaving 4,633 unique records for screening. Several search terms were deliberately broad (“colostomy”, “gracilis flap”, “stromal vascular fraction”), and the majority of retrieved records were excluded on the basis of the title alone as clearly unrelated to rectovaginal fistula. Screening decisions were not documented at the level of individual records, and the number of full texts assessed and the reasons for exclusion at that stage therefore cannot be reconstructed retrospectively. Fifty-one publications were included from the primary search, and a further nineteen, all published before 2020, were identified through screening of reference lists, giving a total of seventy cited publications. This figure is the number of publications cited, not the size of the clinical evidence base, which is smaller and is defined below. Of the seventy included publications, twenty-six are primary clinical studies, systematic reviews or meta-analyses reporting outcomes of the interventions under review; these are summarised individually in **Supplementary Table 1**. The remaining forty-four were included for anatomical, radiotherapeutic, methodological or background context – comprising dose–effect and late-toxicity data, anatomical and technical descriptions, regenerative-medicine mechanism studies, stoma-related reviews, and outcome instruments – and are cited in the text at the point of use rather than tabulated. The selection process is summarised in **Figure 1**.

## 3 Results

The following sections present a narrative synthesis of the retrieved literature. No quantitative pooling was undertaken; all pooled estimates quoted are those reported by the original authors.

### 3.1 Pre-operative assessment

#### 3.1.1 Diagnostic workup and exclusion of active malignancy

In patients presenting with a suspected rectovaginal fistula, clinicians must consider a broad differential diagnosis that encompasses obstetric trauma, Crohn’s disease, radiation injury, or malignancies of the anorectum, perineum, and gynecological organs (10). Establishing a definitive diagnosis and identifying the underlying pathology is a critical initial step (10, 11). Because anorectal inflammatory disease may be secondary to or associated with underlying neoplasia, the presence of a soft-tissue mass, focal luminal wall thickening, or suspicious regional lymphadenopathy on pelvic evaluation should immediately raise concern for a malignant process (10).

A comprehensive pre-operative diagnostic workup should comprise:

Tissue biopsy. Performing histopathological examination of tissue samples retrieved from suspicious lesions is crucial to definitively verify or exclude the presence and origin of an underlying malignancy (11).

Endoscopic evaluation. Direct mucosal visualization via colposcopy, rigid proctosigmoidoscopy, or flexible endoscopes enables precise localization of the fistulous orifice, assessment of coexisting tissue pathology, and identification of synchronous colorectal or gynecological neoplasms (11).

Cross-sectional pelvic imaging (MRI and CT). Intravenous contrast-enhanced pelvic MRI serves as an exceptionally reliable tool for delineating complex fistula architecture, assessing the sphincter complex, mapping the relation to the dentate line, and discriminating active hypervascular inflammatory tracts from chronic radiation fibrosis (10, 12). However, imaging findings must be interpreted strictly as an adjunct to thorough clinical examination, as false-positive imaging results can occur (12). Pelvic CT with intravenous or water-soluble luminal contrast represents a valuable complementary modality, particularly for identifying associated pelvic fluid collections, soft-tissue masses, or structural fistulous communications (10).

Therapeutic implications. The radiological or histological confirmation of active malignancy fundamentally redirects clinical strategy away from local reconstructive interventions toward oncologic management and symptom-relieving measures (10). Consequently, definitive reconstructive surgery must be reserved strictly for cases with clear, multi-modal confirmation of non-malignant disease (10, 12).

#### 3.1.2 Assessment of the fistula, the irradiated field and the patient

Examination under anaesthesia. Where clinical assessment in the outpatient setting is limited by pain or by the extent of fibrosis, examination under anaesthesia allows accurate determination of fistula size, level and complexity, its relation to the anal sphincter, and the presence of rectal or vaginal stricture.

Assessment of the irradiated field. The severity of radiation proctopathy and of vaginal tissue injury should be documented, as both determine the feasibility of local repair. Chronic radiation proctopathy affects a substantial proportion of patients treated for pelvic malignancy and may coexist with stricture (13). Vaginal stenosis is a further late effect of the index radiotherapy, with a two-year actuarial rate of grade ≥ 2 stenosis of 21% in a prospective cohort of 630 patients (14).

Patient-related factors. Baseline continence and sphincter function should be assessed before any reconstructive plan is made, since a technically successful repair in a patient with pre-existing incontinence may not restore function. The number and nature of previous repair attempts is among the strongest determinants of outcome: 98% of patients in one gracilis series and 93% in another had undergone at least one failed repair, with a mean of 3.6 and 2.4 procedures respectively (15, 16). Nutritional status, anaemia, smoking and comorbidity should be optimised before surgery (17). The interval since radiotherapy should be recorded, since it distinguishes radiation-induced from anastomotic fistula and bears on the state of the tissue bed; medians of 8.9 to 20 months have been reported, with a range extending to 240 (6, 18).

Active infection. Pelvic infection or abscess should be controlled before reconstruction is attempted; postoperative perineal infection was one of only two factors associated with failure after graciloplasty in a bicentric series (15).

Multidisciplinary assessment. Given the number of variables involved, assessment involving gynaecologic oncology, colorectal surgery, reconstructive surgery, radiology and radiation oncology is advisable, although this recommendation rests on the complexity of the clinical problem rather than on comparative evidence.

### 3.2 Radiotherapeutic determinants of fistula formation

Rectovaginal fistula (RVF) in women treated for pelvic malignancy is not a uniform entity, and the reconstructive problem differs according to aetiology. In gynaecologic oncology the dominant mechanism is progressive radiation-induced endarteritis obliterans with ischaemic necrosis of the rectovaginal septum, and risk relates to the cumulative external-beam and brachytherapy dose delivered to the anterior rectal wall rather than to either component in isolation. In the prospective multicentre EMBRACE study, a rectal ${\text{D}}_{2{\text{cm}}^{3}}$ of 75 Gy EQD2 or above carried a 12.5% three-year risk of fistula, against 0–2.7% at lower doses (19). Pooled EMBRACE-I data confirmed rectal ${\text{D}}_{2{\text{cm}}^{3}}$ and the ICRU rectovaginal reference point as determinants of grade ≥ 3 anorectal events (20). Concurrent platinum-based chemoradiation constitutes the baseline exposure in these cohorts, whereas subsequent antiangiogenic therapy markedly amplifies risk: in GOG-240, fistula of any grade occurred in 15% versus 1%, in both arms exclusively in previously irradiated patients (21). Prior pelvic surgery further compromises the tissue bed – radical hysterectomy preceded radiotherapy in 54% of one dedicated RVF series, and five of six patients developing fistula on bevacizumab had undergone pelvic surgery in addition to irradiation – as does salvage re-irradiation, after which severe late complications including fistula reached 24.3% in a contemporary image-guided series (6, 22, 23). Latency is discriminating: medians of 8.9 to 20 months after radiotherapy, with a range extending to 240 months, against a median of 19.5 days after low anterior resection, where distance from the anal verge and anastomotic quality predominate (6, 18, 24). Reconstruction in this population therefore proceeds within irradiated, poorly vascularised tissue, which underlies the emphasis on interposition of non-irradiated tissue in the techniques reviewed below.

### 3.3 Treatment options

#### 3.3.1 Martius flap

Irradiated tissue is characterised by poor blood supply, significant fibrosis, and increased tension, which promotes the formation of fistulas. At the same time, they have limited healing potential, which is significantly associated with a lower success rate (1, 25). The use of the Martius flap in radiation-induced fistulas is of particular value because it provides well-vascularized tissue, promotes neovascularization, and effectively separates the vaginal and rectal suture lines, thereby minimizing the risk of recurrence (1, 26). Due to the complex nature of radiation-induced fistulas, treatment is often multi-stage. Definitive repair is generally deferred until the irradiated tissue has stabilised, although the optimal interval has not been established. Faecal diversion before Martius flap repair is widely practised: in the largest dedicated series all 24 patients were diverted, either before the procedure or at the time of surgery (27). An additional proposed strategy is the combination of this procedure with the administration of autologous microfragmented adipose tissue (MFAT), enriched with mesenchymal stem cells (MSCs). MSCs, as multipotent stem cells, have the ability to differentiate into various cell types and exhibit a range of regenerative properties, including immunomodulatory, anti-inflammatory, anti-apoptotic, antioxidant, angiogenic, and analgesic effects. These properties provide a biological rationale for combining the flap with MFAT, although clinical experience is currently limited to a single case description (28). In this case, the Martius flap serves as a vascularized scaffold, while the additional administration of MFAT supports healing through its properties, which may prove beneficial in areas with impaired blood supply and chronic inflammation (26, 28).

Surgical procedures using the Martius flap, in both the classic and modified methods, are based on the same reconstructive principles, although the modified Martius flap does not include a muscular component. The fistula tract is generally excised, and the defect in the rectal wall is closed, most often with single-layer sutures. Concomitant sphincteroplasty may be considered where the sphincter complex is involved. Next, a Martius flap is prepared to cover the defect and reinforce the repair site, thereby creating optimal conditions for healing (26, 29, 30). After excision of the fistula tract and closure of the defect in the rectal wall, the next step is to prepare a vertical incision on the labia majora at a location that allows for the harvest of a vascularized adipose tissue flap of sufficient length to reach the rectovaginal space. The flap may be raised on either pedicle. An anterior pedicle has been described for urogynaecological indications and a posterior pedicle for colorectal repair including RVF, the choice being determined by the reach required rather than by a fixed rule (29, 31). Next, a subcutaneous tunnel is created beneath the bulbocavernosus (bulbospongiosus) muscle, through which the flap is advanced toward the reconstruction site, avoiding twisting or excessive tension on the flap to prevent disruption of microcirculation. Once positioned in the rectovaginal space, the flap is stabilized with sutures to the surrounding tissues, which ensures separation of the vaginal and rectal suture lines, fills the dead space, and improves conditions for neovascularization. The procedure concludes with closure of the incision in the perineum and labia, often with placement of a drain at the flap harvest site (26, 32). Perioperative antibiotic prophylaxis is recommended according to institutional protocols to reduce the risk of surgical-site infection (1, 33).

The Martius method has been reported to achieve high closure rates in selected patients in the treatment of complex and recurrent RVFs. It offers several advantages in fistula repair: acceptable cosmetic outcomes, the need for only one surgical field, and minimal recovery time; however, there are also several adverse effects: postoperative dyspareunia or paroxysmal anal pain (proctalgia) (26, 27, 32). In the systematic review and proportional meta-analysis by Swindon et al., 11 retrospective and 1 prospective study comprising 137 Martius flap procedures were analysed: 44 primary and 93 recurrent fistulas, in a cohort of mean age 42.4 ± 15.7 years. Success was defined as complete fistula healing, although definitions varied across the included studies. A protective stoma was used variably, and four of the twelve studies used none. Follow-up ranged from 3 to 128 months across studies and was not pooled. Closure was achieved in 91.4% (95% CI 79.5–98.5; I^2^ = 32.1%) of primary and 77.5% (95% CI 62.2–89.7; I^2^ = 58.1%) of recurrent fistulas, with an overall recurrence rate of 12.0% (95% CI 5.0–21.9) and a complication rate of 29% (95% CI 9.0–54.7; I^2^ = 85.4%). In the radiation-induced subgroup closure reached 94.6% (95% CI 83.3–99.8; I^2^ = 0%), but this estimate derives from only 26 procedures reported in two series published in 1982 and 1988, before the introduction of image-guided adaptive brachytherapy. Risk of bias was assessed with ROBINS-I: seven of twelve studies were at serious and five at moderate risk, and none at low risk. Dyspareunia was reported in 6 of 137 procedures (4.5%), although only two of the twelve included studies applied objective measures of sexual function (26). Platelet-rich plasma has also been described as an adjunct to Martius flap repair. It is a source of growth factors, adhesion proteins and chemotactic mediators involved in angiogenesis and tissue regeneration, although the reported experience concerns vesicovaginal rather than rectovaginal fistula (25).

Exclusion criteria may vary depending on specific clinical protocols; however, the most commonly cited limitations include: the location of the fistula, the size of the defect, active inflammation, and the patient’s general condition. The method is most often described for low RVFs with defects no larger than 1.5 cm in diameter, although this threshold reflects reported selection practice rather than a validated cut-off; larger defects have been repaired successfully, with a mean diameter of 2.25 ±,0.62 cm and a maximum of 3.5 cm in one series (26, 27, 33).

#### 3.3.2 Graciloplasty

Gracilis muscle interposition is one of the surgical methods which has been used in selected patients with radiation-induced fistulas, particularly in cases of complex fistulas – including those resulting from radiation therapy – which are characterised by a high rate of recurrence and postoperative complications (15, 34). This method is recommended as a subsequent attempt to close the fistula, particularly in cases of recurrent RVFs, but due to the surgical complexity, it is recommended as a secondary repair following failed primary interventions (7, 15).

The gracilis muscle is a well-vascularized adductor muscle of the thigh. Its main vascular pedicle most commonly (in 94.4% of cases) originates from the deep femoral artery. Less commonly, it may originate from the superficial femoral artery (2.8%) or the medial circumflex femoral artery (2.8%). Distal vascular pedicles are present, ranging in number from one to four, with two distal pedicles being the most common (46.9%). They originate from the superficial femoral artery or, in more distal segments, from the popliteal artery. An additional proximal pedicle is present in 28.9% (34–37). Innervation of the muscle is provided by a branch of the obturator nerve (36).

During flap harvesting, the patient is positioned in the supine-lithotomy position, and the lower limb is abducted and externally rotated. In this position, the adductor longus is easily palpable at the beginning of its course and defines an approximate line connecting the pubic bone and the medial aspect of the knee. The gracilis muscle is located posterior to the origin of the adductor longus, approximately 2–4 cm from the designated line. Nerves and vessels are located 10–15 cm away. Two or three incisions are made at the anticipated location of the gracilis muscle. After isolating the muscle, it is meticulously dissected to identify the vessels, and then its distal end is pulled through a prepared tunnel between the proximal incision and the perineum (34, 36, 37).

In the case of an RVF, the defect is initially excised to obtain a margin of healthy tissue and closed using absorbable sutures. The harvested flap is then placed between the vagina and the rectum to reinforce the rectovaginal septum, allowing the tissue healing process to begin. The perineal wound is closed externally; after examining the vagina, several additional sutures are placed to secure the flap to the vaginal wall (15, 35). If the patient also had problems with the anal sphincter, the muscle can be looped around the anal canal, thereby reconstructing the sphincter (37).

Three essential steps contribute to success: the tunnel must be wide enough to allow for tension-free muscle positioning; positioning the muscle at or slightly above the excised fistula is essential for healing; and perineal incisions should be avoided, as wound healing in this region may be impaired (16). Intraoperative perfusion assessment using indocyanine green near-infrared fluorescence (ICG-NIRF) has been proposed as a technical refinement. After mobilizing the gracilis muscle prior to its final placement in the perineal region, the patient is administered indocyanine green (ICG) intravenously, which initially remains in the intravascular space, and its near-infrared fluorescence signal is proportional to perfusion. This replaces subjective intraoperative parameters such as tissue colour and palpable pulse with a quantifiable measure, and the derived perfusion values correlated with clinical outcome in the reported series. Whether this translates into higher closure rates has not been evaluated (38).

When evaluating success rates for graciloplasty, it is essential to distinguish between general complex fistula cohorts and the radiation-induced RVF population. Results from the meta-analysis by Garoufalia et al., including 658 patients from 25 studies, were evaluated. RVF was the largest subgroup (50.7%), but the overall analyzed population was heterogeneous, including rectourethral and other complex perineal fistulas. A history of radiotherapy was reported in approximately 18% of patients. The study reported the weighted mean success rate of the procedure was 79.4% (95% CI 73.8–85.0) with complete RVF healing achieved in 74.9% (95% CI 67.0–82.9) of patients in the complex fistula subgroup. Success after the index procedure alone was 62.8% (95% CI 40.5–85.2), rising to 84.5% (95% CI 74.1–95.0) once additional procedures were counted. Following a follow-up period of less than 2 years, fistula healing was observed in 82.3% (95% CI 74.5–88.7) of patients, and in 75.1% (95% CI 66.8–82.3) of patients after more than 2 years. The overall rate of fistula recurrence was 16.7% (95% CI 11.0–22.3) and short-term postoperative complications occurred in 25.7% (95% CI 18.1–33.2). Treatment success was not uniformly defined across the included studies; in most it denoted absence of discharge of faeces, flatus or mucus through the fistula without a diverting stoma. Twenty-four of the twenty-five studies were at moderate and one at low risk of bias, and the authors graded the certainty of evidence for success, complications and recurrence as very low, with significant publication bias detected for both (Egger’s test p=0.001). This study provides indirect evidence supporting the use of vascularized gracilis interposition in complex fistulas, particularly in poorly vascularized or irradiated fields, but cannot be considered direct evidence (34).

Functional and quality-of-life outcomes were not assessed in the meta-analysis, and where individual series have reported them the results diverge: sexual function deteriorated in 79% in one cohort (39) and improved significantly in another (40). Schoene et al. reported long-term outcomes of gracilis muscle transposition in 60 patients with complex anorectal fistulas of heterogeneous etiologies, with a median follow-up period of 35.9 months (range 1–187). The initial success of this method was estimated at 33.3% (95% CI 22.5–46.3), and with additional interventions, ultimately at 65% (95% CI 52.4–75.8), where RVFs represented the primary subgroup, accounting for 58.3% of cases (35/60; 95% CI 45.7–70.0). Most patients (98%) had already undergone previous treatment attempts. Outcomes were reported separately by aetiology: among 17 patients with radiation-induced fistula, primary healing was achieved in 6 (35.3%) and final healing in 8 (47.1%) – the lowest of the four aetiological groups, compared with 87.5% after obstetric injury and 76.2% for iatrogenic causes (16).

Additionally, Oner et al. (2025) reported the use of gracilis muscle flap reconstruction in a heterogeneous cohort of patients with complex pelvic fistulas and perineal defects with a median follow-up of 24 months. The authors emphasized the significant role of a temporary protective stoma as a factor influencing treatment outcomes using this method. Although most patients had a history of previous radiation therapy (72.2%; 13/18), the study was not specifically designed to evaluate radiation-induced rectovaginal fistulas (35).

In a retrospective study, Corté et al. analysed 286 procedures performed in 79 patients with rectovaginal fistula, including 32 graciloplasties. After a mean follow-up period of 33 months, the overall success rate was 72% (57/79). Multivariable analysis identified more extensive surgical procedures and use of a temporary stoma as independent predictors of success, whereas patient age and fistula aetiology were not (41). Frontali et al. analyzed 61 cases of graciloplasty for RVFs and demonstrated that treatment failure (persistence of the fistula and/or permanent stoma) occurred in 39% (24/61) of patients. The mean follow-up period was 56 ± 48 months (1–183 months), allowing assessment of long-term outcomes. In this study, which aimed to identify risk factors for this method in cases of RVFs, two factors were identified: the absence of postoperative antibiotic prophylaxis and the occurrence of postoperative perineal infection (15). These two studies evaluated graciloplasty across diverse etiologies, identifying temporary fecal diversion and absence of perineal infection as key prognostic factors rather than etiology alone (15, 41).

The most common complications that may occur with this method include: surgical site infections, impaired wound healing, incisional hernia, scar tissue pain, paresthesia of the thigh or the entire lower limb, and necrosis of the gracilis muscle; long-term complications include vaginal strictures, urinary or fecal incontinence, and prolonged numbness of the thighs (16, 34). Careful patient selection based on fistula location and oncological status is critical when considering graciloplasty. In some centers patients were excluded due to the high location of the RVF, as these fistulas are more commonly treated via an intra-abdominal approach. Similarly, active pelvic malignancy represents a major contraindication to reconstructive surgery. In such cases, a diverting ostomy was performed instead of gracilis muscle interposition (40).

In the non-radiation RVF population, a traditional stepwise approach is generally recommended, starting with local treatment and reserving graciloplasty for re-interventions following failed attempts (15, 34). In the context of radiation-induced rectovaginal fistulas, several authors advocate for a more proactive, early application of graciloplasty. This aggressive approach is justified by the extensive severe fibrosis and tissue ischaemia characteristic of irradiated fields (7, 16, 41). Closure rates reported for graciloplasty vary widely, from 33.3% for the index procedure in a cohort in which 98% of patients had failed previous repairs, to a weighted mean of 79.4% across pooled series of complex perineal fistulas (16, 34). This range reflects differences in aetiology, number of previous repairs, diversion practice and definition of success rather than differences in the technique itself, and it precludes direct comparison between series. Significant challenges in performing this type of surgical procedure include large tissue defects, previous failed repairs, fibrosis, poorly vascularized surrounding tissues, and radiation-induced tissue damage, all of which adversely affect the final outcome (16, 42). A factor that may potentially increase success rates is a temporary stoma, as most patients are already fitted with one prior to the procedure or have it created during the surgery (15, 16, 35). The method improves overall patient quality of life, but clinicians must be careful that the procedure can cause persistent sexual dysfunction due to postoperative dyspareunia (39, 40).

In the event of failure of the initial surgical treatment, reoperation is possible, with a secondary healing rate of 58% reported by Muller et al. in patients undergoing reoperation (43). Alternative techniques under consideration include: delayed colo-anal anastomosis (DCAA), local repair via transanal approach, stoma creation, and repeat ileo-anal anastomosis (Redo IPAA), as well as repeat graciloplasty; however, in the study described, it failed in all cases. In the context of recurrent RVFs following failed graciloplasty, due to the very low success rate of conservative repairs, it is recommended to consider DCAA earlier (43).

#### 3.3.3 Colostomy

Due to impaired healing and tissue fibrosis in cases of radiation-induced fistulas, surgical reconstruction is often not feasible. Additionally, most patients with RI-RVF are elderly and often have comorbidities, which can significantly compromise treatment outcomes (6, 17). Therefore, faecal diversion via colostomy or diverting ileostomy is often an appropriate initial, bridging or palliative management strategy in patients with radiation-induced rectovaginal fistula; comparable considerations regarding the role and avoidance of diversion have been described for complex anal fistulas, though that population differs from RI-RVF (5, 6, 44). A distinction should be drawn between temporary diversion, established to control symptoms and prepare the tissue bed before planned reconstruction, and permanent palliative diversion, chosen as definitive management where reconstruction is not feasible or not desired.

The purpose of creating a stoma is to divert stool away from the anus, thereby preventing infection and mechanical trauma to the fistula site caused by fecal matter. This reduces the risk of sepsis and improves the healing process, while also enhancing patients’ quality of life (44, 45). A stoma can be used as a temporary solution, allowing tissue to heal and preparing the surgical site for possible future reconstruction, or as a definitive, palliative solution that improves patients’ quality of life. However, the choice of treatment should be individualized and depends on the anatomy of the fistula, the extent of radiation-induced damage, the presence of an active infection or tumor recurrence, comorbidities, sphincter function, previous attempts at surgical treatment, and the patient’s preferences (6, 17, 45).

Colostomy alone can reduce symptoms such as pain, bleeding, perineal discharge, and urgency, but it does not eliminate them completely and offers little chance of spontaneous closure of the radiation-induced RVF. In a study conducted by Zelga et al. involving 50 patients with RI-RVF, of whom 48 (96%) underwent fecal diversion alone, it was shown that spontaneous healing of the fistula occurred in only 6 patients (12%) (6). No series reporting diversion alone assessed continence, sexual function or quality of life. After the fistula has healed, an attempt can be made to close the stoma; however, good functional outcomes have been achieved in only a few cases. In a series of patients with RI-RVF undergoing restorative resection, stoma reversal was achievable in only 30% of cases (2).

The incidence of complications ranges from 10% to 70% in the literature. Early complications, occurring within the first 30 days, include hematoma, ischaemia, necrosis, stoma retraction, and peristomal abscesses. Late complications include stoma prolapse, stoma stricture, peristomal hernia, retraction, intestinal obstruction, varicose veins, fluid and electrolyte imbalances, and bleeding (46, 47). In a meta-analysis of preventive diversion in colorectal surgery – a population distinct from RI-RVF, so that these findings are indirect – no significant differences were found between ileostomy and colostomy for most complications, with the exception of stoma prolapse, which occurred more frequently with colostomy. Ileostomy was more often associated with skin irritation, peristomal hernia, dehydration and infection, whereas colostomy more often led to fistula, stricture and bleeding. The two stoma types therefore carry distinct complication profiles and require correspondingly different monitoring (48). These complications can significantly impact patients’ quality of life. Early pre- and postoperative education covering both technical and psychosocial aspects is crucial for reducing the risk of complications and improving the patient’s adaptability to life with a stoma (46–48).

Zhen Liu et al., in a study comparing laparoscopic and open colostomy in patients with RI-RVF, demonstrated that laparoscopic surgery is feasible in patients with RI-RVF and does not increase the incidence of stoma-related complications or postoperative morbidity, but is associated with a shorter hospital stay and a lower risk of surgical site infections compared to the open procedure (5).

There are no absolute contraindications to ostomy creation. However, the literature identifies relative contraindications, such as peritoneal carcinomatosis or a short mesentery that prevents tension-free bowel emergence. Excessive stoma tension, which is more common in obese individuals, is an independent risk factor for complications (46).

#### 3.3.4 Coloanal anastomosis

Coloanal anastomosis is a procedure involving the resection of a segment damaged by radiation therapy and the anastomosis of the colon to the anus using healthy tissue to restore the continuity of the gastrointestinal tract (2). The main variations of this procedure are immediate coloanal anastomosis (ICAA), often performed using the Parks method, and delayed coloanal anastomosis (DCAA), performed using the Turnbull-Cutait method (49–51).

The Parks immediate coloanal anastomosis involves excising the diseased rectal mucosa along with its upper portion and a segment of the sigmoid colon. A muscular cuff is left in place, into which an unaffected segment of the large intestine is inserted using the pull-through technique, followed by anastomosis of the colon to the anus. The procedure requires securing with a proximal colostomy, which can be closed 3 months after the procedure (49).

Another described method is the Turnbull-Cutait procedure, i.e., an abdominoperineal resection with delayed coloanal anastomosis. The essence of this method is performing the coloanal anastomosis in two temporally separated stages. In the first stage, a rectal resection is performed, and then 5–10 cm of the colon is brought out through the anus and left outside the body. After 7–10 days, the second stage is performed, in which the exposed segment of the colon is transected, followed by the actual anastomosis. The time interval between stages allows for the formation of adhesions between the colon and the anal canal, which reduces the risk of anastomotic leakage and the development of an anastomotic fistula. The two-stage approach yields better outcomes in patients with radiation-induced tissue damage and offers a greater chance of restoring gastrointestinal function without the need for a permanent colostomy (50, 51).

Surgical management carries a risk of complications such as low anterior rectal resection syndrome, but is associated with a higher rate of symptom remission and a greater chance of restoring intestinal continuity than colostomy alone. Most patients who undergo surgery achieve good results on functional and symptom scales. Studies confirm a significant improvement in quality of life following resection of the damaged intestine, regardless of whether the stoma was reversed or maintained (2, 49, 51).

The comparative evidence for DCAA versus ICAA derives almost entirely from low rectal cancer surgery rather than from populations with radiation-induced rectovaginal fistula, and its applicability to this setting is therefore indirect (52–54). Two meta-analyses have compared the techniques in low rectal cancer. La Raja et al., restricting the quantitative synthesis to four comparative studies, found a significantly lower rate of pelvic sepsis with DCAA (7% vs. 14%; OR 0.37, 95% CI 0.16–0.85, p=0.02) but no difference in Clavien–Dindo ≥ III complications (OR 1.17, 95% CI 0.38–3.62) or in the risk of a definitive stoma (OR 0.77, 95% CI 0.15–3.85) (52). Wang et al., pooling 16 studies and 1,409 patients, found significantly higher risks in the ICAA group for total complications (OR 2.74, 95% CI 1.89–3.98), anastomosis-related complications (OR 3.46, 95% CI 2.32–5.15) and anastomotic leakage (OR 2.79, 95% CI 1.71–4.57), with no difference in operative time, blood loss, length of stay, or oncological outcomes (53). In a retrospective study involving 305 patients (including 109 in the DCAA group and 196 in the ICAA group), it was shown that the incidence of complications in the DCAA group was lower than in the ICAA group (15% vs. 31%, p=0.002); early and late anastomotic complications were also observed less frequently in the DCAA group than in the ICAA group, 7% vs. 15% ( p=0.047) and 2% vs. 11% ( p=0.005), respectively. Two years after surgery, the DCAA cohort reported a lower percentage of patients with LARS (38% vs. 60%, p=0.018) and severe fecal incontinence (0% vs. 8%, p=0.029) than the ICAA group (54).

DCAA may be an effective treatment for complex rectovaginal fistula resulting, among other causes, from pelvic radiation therapy. In the GRECCAR study, among the analyzed cohort of 78 patients, 49% had a history of prior pelvic radiation therapy, and 10% had a radiation-induced fistula. DCAA resulted in fistula healing with preserved bowel continuity in 81% (63/78). Prior pelvic radiotherapy did not increase the risk of failure (p = 0.331). Morbidity was nonetheless substantial: overall 45%, major 23%, anastomotic leakage 17%, with one postoperative death; and despite technical success, 42% (24/57) of assessed patients had major LARS or required a stoma for poor bowel function. It should be emphasized, however, that the treatment of RVF following radiation therapy remains a major challenge due to impaired healing, an increased risk of complications, and the possibility of postoperative functional disorders. DCAA therefore offers a realistic prospect of restoring continuity in appropriately selected patients, at the cost of substantial morbidity and functional sequelae that should be discussed before surgery (55).

The results suggest that DCAA may be particularly beneficial for patients who cannot tolerate a stoma or for patients with impaired tissue healing and a high risk of anastomotic leakage, including those with radiation-induced tissue damage (50–52, 54, 55).

#### 3.3.5 Stromal vascular fraction

The stromal vascular fraction (SVF) is a heterogeneous population of cells derived from adipose tissue, comprising pericytes, smooth muscle cells, immune cells, and adipose-derived stem cells (ADSCs). By secreting cytokines, growth factors, and exosomes, it promotes angiogenesis, tissue regeneration, collagen synthesis, and the reduction of inflammation, making it a promising tool in regenerative medicine (56–59). Trevor et al. demonstrated that irradiation does not reduce the viability and functionality of stem cells present in SVF, suggesting their potential for use in the treatment of radiation-induced wounds (56).

Preparation methods differ substantially between reports. Enzymatic isolation with collagenase yields a higher cell count per unit volume of adipose tissue than mechanical methods, but requires dedicated equipment, consumables, trained laboratory personnel and conditions for aseptic transport, which restricts its use to specialised centres. The cellular composition of the resulting suspension is variable: mesenchymal stromal cells are reported to constitute between 10% and 20% of the fraction, and in one detailed characterisation the preparation contained 9.7% CD44+ and 10.2% CD90+ cells (13). Administered volumes and techniques also differ, ranging from 3 mL injected beneath a vaginal mucosal flap to staged cell-assisted lipotransfer combining 5 mL of SVF with 28–30 mL of fat graft, and endoscopic submucosal injection through a 0.7 mm needle (13, 60). Outcome definitions vary correspondingly, from absence of clinical recurrence to radiological and histological confirmation of healing.

Kim et al. described two patients who developed rectovaginal fistula after chemoradiotherapy and low anterior resection for rectal cancer. In the first, SVF was injected beneath a vaginal mucosal flap; in the second, two staged cell-assisted lipotransfer procedures were performed. Both fistulas closed without recurrence at 12 and 5 months respectively (60).

In a separate case report, submucosal SVF injection reduced inflammation and promoted mucosal regeneration in refractory chronic radiation proctitis (13). Taken together, clinical experience with SVF in irradiated pelvic tissue is confined to two case reports comprising three patients: two with a rectovaginal fistula arising after chemoradiotherapy and low anterior resection for rectal cancer, and one with refractory chronic radiation proctitis without fistula (13, 60). In none of them did the lesion follow radiotherapy for gynaecological malignancy, and no comparative study exists. Whether SVF behaves comparably in a septum damaged by primary radionecrosis, rather than by anastomotic breakdown in a previously irradiated field, is unknown. Neither case report assessed functional or patient-reported outcomes.

Despite its great potential in regenerative medicine, SVF therapy has several significant limitations. The heterogeneity of the preparations, the lack of full standardization, and limited knowledge of the mechanisms of action contribute to variability in therapeutic outcomes (61–64). Manufacturing variability compounds this problem: no standardised protocol exists for isolation, cell counting or release testing, and preparations described under the same name may differ substantially in composition.

Additionally, efficacy depends on patient characteristics, and there is still a lack of long-term data on safety and the durability of the effects. Another significant limitation is the regulatory framework governing cell manipulation and the preparation of cell-based products, which restricts access to this therapy. The potential risk of stimulating the tumor stroma is also being considered, although current data do not allow for an unambiguous assessment of this phenomenon. Furthermore, the available scientific evidence is based primarily on case reports and small clinical series, which limits the ability to draw definitive conclusions. For this reason, SVF therapy requires further research and standardization of procedures (58, 59, 61–64).

### 3.4 Proposed management framework

**Figure 2:**
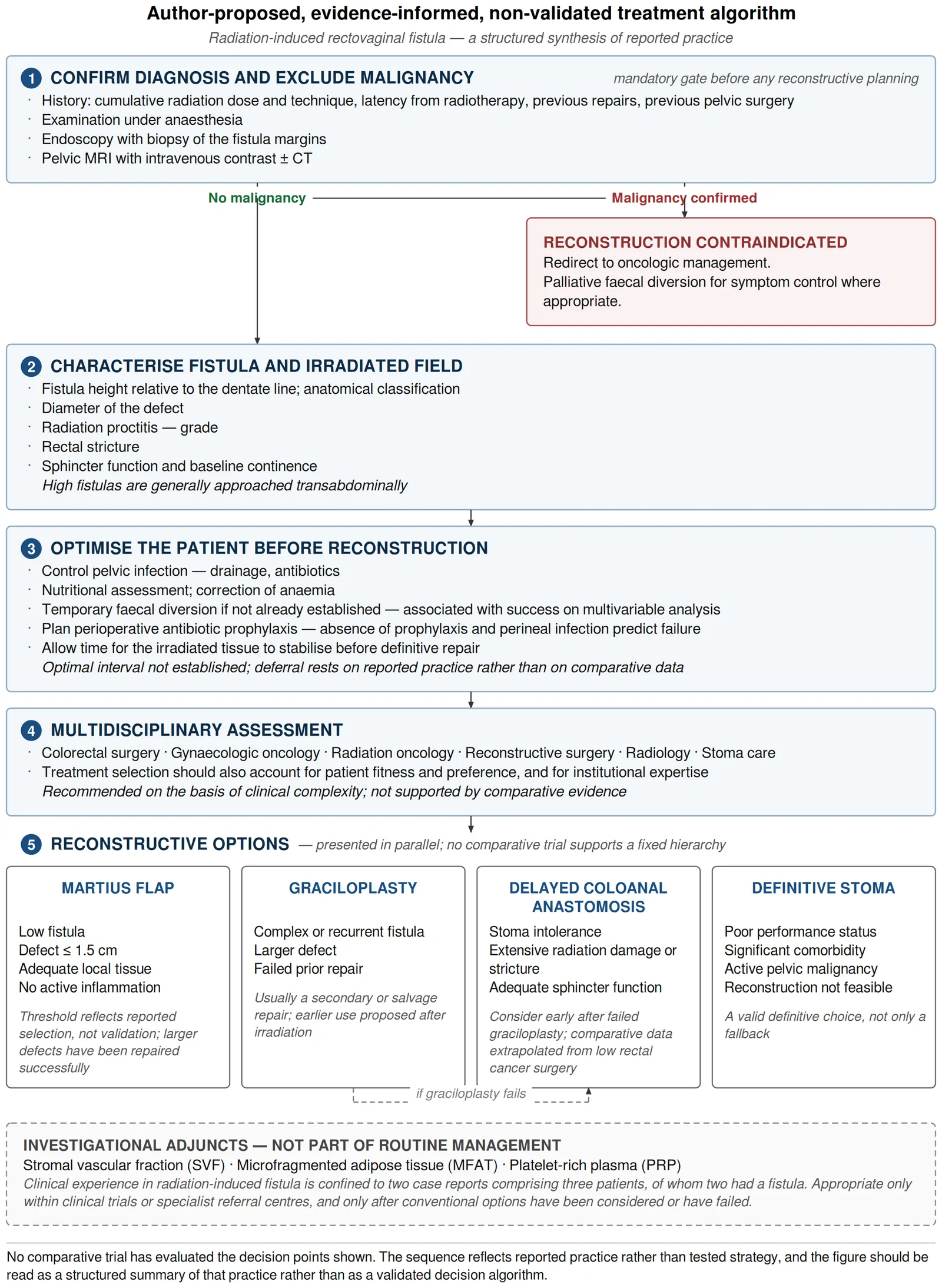
Author-proposed, evidence-informed, non-validated treatment algorithm for radiation-induced rectovaginal fistula. No comparative trial has evaluated the decision points shown. Exclusion of malignancy and the diagnostic work-up in step 1 follow (10–12); optimisation of the patient in step 3 follows (17). Among the reconstructive options, Martius flap repair is supported by (26, 27, 33), gracilis interposition by (15, 16, 34), delayed coloanal anastomosis by (50, 51, 55) and definitive stoma by (5, 6, 45); the investigational adjuncts by (13, 56, 60). The 1.5 cm threshold for Martius flap repair derives from reported selection criteria (26, 33) and has not been prospectively validated; larger defects have been repaired successfully, with a mean diameter of 2.25 cm and a maximum of 3.5 cm in one series (27). Above this threshold no size-based cut-off can be defined, and larger defects are approached according to fistula height, tissue quality and previous repairs rather than by diameter. Definitive reconstruction is deferred until the irradiated tissue has stabilised; the optimal interval has not been established. Stromal vascular fraction is shown as an investigational adjunct only.

The framework summarised in **Figure 2** synthesises the treatment sequences described across the reviewed literature (1, 4, 6, 16, 17, 26, 34, 49, 51, 55). Several qualifications are necessary. No comparative study has evaluated the decision points it contains, and the sequence reflects reported practice rather than tested strategy. Exclusion of persistent or recurrent malignancy by biopsy is the only step supported unambiguously, since confirmation of active disease redirects management away from reconstruction entirely and towards oncologic treatment or palliative diversion (10–12). The 1.5 cm threshold for Martius flap repair reflects selection criteria applied in published series rather than a validated cut-off, and no comparable threshold can be defined for graciloplasty or delayed coloanal anastomosis, where reported cohorts are heterogeneous in aetiology, prior treatment and diversion practice (16, 26, 33–35, 55). The choice between these techniques currently rests on fistula height, local tissue quality, the number of previous repairs, and surgeon experience, rather than on any measurable parameter (16, 34, 40, 42). In the only multivariable analysis addressing this question, the extent of the procedure and the use of a temporary stoma predicted success, whereas patient age and fistula aetiology did not – although with only four radiation-induced fistulas in that cohort, the analysis had little power to detect an effect of irradiation specifically (41). Several determinants that materially affect feasibility are captured inconsistently or not at all across the published series: the grade of radiation proctitis, the presence of rectal stricture, sphincter function and baseline continence, active pelvic infection, nutritional status and anaemia, the cumulative radiation dose delivered to the anterior rectal wall, and the number of previous repair attempts (4, 8, 15–17, 19, 20, 37, 44, 51). None of these has been evaluated as a predictor of reconstructive outcome in a prospective design. Multidisciplinary assessment involving colorectal surgery, gynaecologic oncology, radiation oncology and stoma care is therefore recommended, though this recommendation rests on the complexity of the clinical problem rather than on comparative evidence (10, 17). Stromal vascular fraction and related regenerative approaches are shown separately as investigational adjuncts, supported at present only by case reports and small series, and should not be regarded as a routine step in management (13, 56, 58–60).

### 3.5 Outcomes beyond anatomical closure

Closure as an endpoint. Anatomical closure has dominated reporting in RI-RVF, yet closure and patient benefit are not equivalent, and closure alone does not capture the burden the condition imposes. In a prospective series of 55 women undergoing RVF repair of mixed etiology, healing after index surgery was 25.5% (14/55) and reached 67.3% (37/55) only after repeated procedures; 18/55 reported pelvic pain and 24/55 vaginal flatulence. The radiation subgroup within that cohort comprised only three patients, illustrating how sparse etiology-specific functional data remain (65). In a separate long-term evaluation of obstetric and cryptoglandular RVF, quality of life and sexual function were comparable irrespective of whether the fistula had healed – a finding that, although derived from non-irradiated patients, directly challenges closure as a sufficient endpoint (66).

Stoma-free survival, stoma reversal and reoperation burden. Frontali et al. defined treatment failure as persistence of the fistula and/or a permanent stoma, which occurred in 24 of 61 patients (39.3%; 95% CI 28.1–51.9) after graciloplasty –in effect a stoma-free survival endpoint, although not named as such (15). After faecal diversion alone, spontaneous closure occurred in 6/50 patients (12%) (6), and following reconstructive resection stoma reversal was achievable in only 30% of cases (2, 6). Corté et al. performed 286 procedures in 79 patients, a mean of 3.6 ± 2.5 interventions per woman, with an overall success rate of 72% (57/79) over a median follow-up of 33 months (41). After failed graciloplasty, salvage reoperation succeeded in 58%, while repeat graciloplasty failed in all cases in one series of 19 consecutive patients (16, 43).

Faecal continence and low anterior resection syndrome. LARS denotes disordered bowel function after rectal resection leading to a detriment in quality of life; the LARS score provides a validated patient-reported instrument, and a meta-analysis of eleven studies applying it reported a pooled prevalence of major LARS of 41% (95% CI 34–48) (67, 68). In an international multicentre comparison of 305 patients (109 DCAA, 196 ICAA), DCAA yielded lower rates of LARS (38% vs. 60%, p=0.018) and of severe faecal incontinence (0% vs. 8%, p=0.029) at two years (54).

Vaginal stenosis. Vaginal stenosis is a late effect of the index radiotherapy rather than of the reconstruction, but it co-determines vaginal function in these women. In 630 patients from the EMBRACE study, the two-year actuarial estimate of grade ≥ 2 vaginal stenosis was 21%, with the rectovaginal reference point dose (HR 1.025, p=0.029), EBRT dose >45 Gy/25 fractions (HR 1.770, p=0.056) and vaginal tumour extension (HR 2.259, p≤0.001) identified as risk factors; modelled risk rose from 20% at 65 Gy to 27% at 75 Gy and 34% at 85 Gy (14).

Pelvic pain. Pelvic pain is seldom reported as an outcome of fistula repair, but is well documented as a late effect of the index radiotherapy. Using a case-finding instrument, 35 of 92 cervical cancer survivors treated with radiotherapy (38%) met criteria for chronic pelvic pain, and intestinal and bladder morbidity remained independently associated with it on multivariable analysis (69). A systematic review confirmed that chronic pelvic pain and bowel morbidity co-occur and jointly reduce quality of life in irradiated cervical cancer patients (70). Within RVF cohorts specifically, pelvic pain was reported by 18 of 55 women in a mixed-etiology series, and proctalgia is a recognised adverse effect of Martius flap repair (26, 65).

Sexual function and dyspareunia. Dyspareunia is reported after both Martius flap and graciloplasty, yet it is rarely quantified. Kersting et al. evaluated 19 women undergoing gracilis transposition for recurrent RVF, with primary healing in 10/19 (53%) and final healing in 14/19 (74%) over a mean follow-up of 23 months. Sexual function deteriorated in 15/19 (79%), was unchanged in three and improved in one; only four women were sexually active before surgery and two afterwards, with pain the main reason for cessation both before and after. Quality of life nonetheless improved in 11/19 (58%), and 15/19 (79%) would undergo the operation again. Donor-site morbidity affected 8/19 (42%) (39). Grott et al. followed 47 transpositions in 46 patients over a 10-year period, of whom 29 had (neo-)rectovaginal fistulas, using the Wexner score, FIQL, SF-12 and the Female Sexual Function Index (42). Picciariello et al. similarly reported functional outcome and quality of life after graciloplasty for complex rectovaginal and rectourethral fistulas (40). In the only cohort providing an explicit denominator, 12 of 47 sexually active women (25.5%) reported dyspareunia at long-term follow-up, although that series comprised obstetric and cryptoglandular rather than radiation-induced fistulas (66). Dyspareunia was reported in 6 of 137 Martius flap procedures (4.5%), although only two of the twelve included studies applied objective measures of postoperative sexual function (26).

Symptom control independent of closure. Symptom relief and anatomical closure diverge in both directions. In a comparative series of 26 women, symptom scores improved in both arms from three months and were sustained to two years, yet no fistula healed spontaneously among the 16 managed with colostomy alone (2). Conversely, in a prospective series applying SF-12 before and after Martius repair, the mental component improved significantly even in women in whom the repair failed or recurred, whereas the physical component did not; the authors attributed this to symptomatic improvement from partial closure and to relief of the psychological burden of the condition (27).

Urinary symptoms. Urinary symptoms are seldom reported. Recurrent urinary tract infection affected 9 of 55 women in one mixed-aetiology series (65).

## 4 Discussion

The management of radiation-induced RVFs remains one of the most daunting challenges in pelvic surgery. As highlighted in the reviewed literature, this condition is not merely a surgical defect but a severe anorectal disorder that has a major impact on quality of life, leading to significant psychological morbidity and social restriction (2, 6–8). Since these fistulas often occur years after radiotherapy for gynecological cancers, clinicians are frequently faced with patients who have dense, inelastic fibrotic tissue and reduced perfusion caused by vascular sclerosis (3, 5). The body’s natural healing capacity is severely limited in such conditions. This means that a universal “gold standard” for treatment has yet to be established, necessitating a highly individualised approach (7). The dosimetric determinants of fistula formation are increasingly well characterised, yet they are almost never reported in the surgical series on which reconstructive practice rests, and this disconnect between the radiotherapeutic and surgical literature is itself an obstacle to progress (19, 20).

For low-lying rectovaginal fistulas with small defects ( <1.5 cm), the Martius flap serves as a valuable reconstructive option by interposing well-vascularized tissue to separate suture lines and mitigate chronic ischaemia (1, 25, 26, 33). However, published efficacy outcomes must be interpreted with substantial caution. Success estimates are predominantly derived from non-randomised, retrospective series characterised by small sample sizes, variable follow-up durations, differing diversion practices, and heterogeneous etiologies. Although pooled data report closure reaching 94.6% in the radiation-induced subgroup, that estimate derives from 26 procedures in two series published before 1990, the pooled complication rate was 29%, and success fell to 77.5% in recurrent fistulas (26). Moreover, patients must be counseled regarding a reported perioperative complication rate of approximately 29%, including dyspareunia and pelvic pain (26). While combining the flap with adjunctive regenerative therapies (e.g. MSCs or PRP) offers a biological rationale, clinical evidence remains preliminary (25, 26, 28).

For complex and recurrent RVFs – particularly in irradiated fields characterised by endarteritis and severe fibrosis – gracilis muscle interposition serves as a valuable option to introduce bulky, well-vascularized non-irradiated tissue (15, 16, 34). However, reported success rates must be evaluated with significant caution due to substantial methodological heterogeneity, mixed fistula etiologies, variable history of prior repairs, and inconsistent stoma diversion protocols across published cohorts (16, 34, 35). In a large meta-analysis by Garoufalia et al. (comprising 658 patients across 25 studies, with RVFs representing 50.7% of cases and approximately 18% having a history of pelvic radiotherapy), the weighted overall success rate was 79.4%, with complete healing achieved in 74.9% of the complex RVF subgroup (34). Nevertheless, this represents indirect evidence, as outcomes were not stratified purely for radiation-induced RVFs. In contrast, cohort studies focusing on heavily pretreated patients demonstrate more modest efficacy; Schoene et al. reported an initial success rate of only 33.3% (increasing to 65% following secondary interventions) in a cohort where 98% had failed prior repairs (16). Furthermore, failure occurred in 24 of 61 patients (39%) in a bicentric series of graciloplasty, with absence of postoperative antibiotic prophylaxis and postoperative perineal infection identified as the only risk factors on univariable analysis; failure fell from 53% without prophylaxis to 14% with it (15). Patients considering graciloplasty must be counseled on a notable perioperative complication rate of approximately 25.7% reported in pooled series, including surgical site infections, muscle necrosis, thigh paresthesia, and long-term dyspareunia (34). Intraoperative innovations such as indocyanine green near-infrared fluorescence (ICG-NIRF) angiography offer an objective assessment of flap perfusion, replacing subjective clinical judgment, though its direct impact on long-term fistula closure rates requires further clinical validation (38).

For many patients, particularly the elderly or those with significant comorbidity, faecal diversion is a reasonable initial or bridging step, and for some it remains the definitive palliative choice (5, 6, 17). It reduces symptoms and the risk of sepsis, but spontaneous closure after diversion alone occurred in only 6 of 50 patients (12%) in the series of Zelga et al. (6). Diversion should therefore be understood as symptom control and tissue preparation rather than as definitive treatment, and the decision between colostomy and ileostomy should take account of their differing complication profiles (48). The benefit of a protective stoma in supporting fistula repair itself remains contested: it was an independent predictor of success in one large series but had no measurable effect on healing in another (41, 66).

Coloanal anastomosis restores bowel continuity in patients seeking to avoid a permanent stoma. The available comparative data favour the delayed over the immediate technique, but not uniformly across endpoints: pooled analyses reported lower rates of anastomotic leakage and pelvic sepsis with DCAA, whereas no difference was found in Clavien–Dindo ≥ III complications or in the risk of a definitive stoma, and the lower rate of major LARS at two years derives from a single retrospective multicentre cohort in which DCAA was performed without a diverting stoma, so that the functional advantage cannot be attributed to the anastomotic technique alone (52–54). This evidence derives almost entirely from low rectal cancer surgery rather than from populations with radiation-induced fistula, and its extrapolation to this setting remains indirect. The two settings differ in ways that matter mechanistically. In rectal cancer surgery the anastomosis is fashioned in tissue that, although irradiated preoperatively, has not undergone years of progressive obliterative endarteritis and fibrosis, and the objective is to avoid a temporary stoma in an otherwise healing patient (52–54). In radiation-induced fistula the tissue bed is chronically hypoperfused, the interval from irradiation is measured in months to years – a median of 20 months in one dedicated series, with a range extending to 240 – and the objective is to close a defect that has already failed to heal. Anastomotic outcomes achieved in the former setting therefore cannot be assumed to transfer to the latter (6).

The GRECCAR cohort remains the most directly relevant evidence for delayed coloanal anastomosis in irradiated patients, although it is a mixed-aetiology series in which only 8 of 78 fistulas were radiation-induced, and only three of those followed radiotherapy for cervical cancer. Prior pelvic radiotherapy was not associated with failure on univariate analysis (29/38 successes, 76%; p=0.331), but this comparison was not adjusted for other factors and the cohort was not powered for it (55).

A recurring limitation of the literature reviewed here is that success is defined almost exclusively as closure of the tract. Closure is a surgical endpoint; it is not necessarily a patient endpoint, and the two diverge more often than the reported success rates suggest. Healing after the index procedure was achieved in a quarter of women in one prospective series and reached two thirds only through repeated intervention, while Corté et al. performed 286 procedures in 79 patients – figures that describe a trajectory of serial operations rather than a single successful repair (41, 65). More strikingly, in a long-term evaluation of obstetric and cryptoglandular fistula, quality of life and sexual function were comparable irrespective of whether the fistula had healed (66). A second consideration is specific to the irradiated patient: a substantial part of the residual burden is attributable to the index radiotherapy rather than to the reconstruction, and is therefore not modifiable by any surgical technique. Vaginal stenosis, chronic pelvic pain and sexual dysfunction all persist independently of the fistula itself (14, 69, 70). A woman whose fistula closes may remain symptomatic from problems that no flap can address. This does not diminish the value of reconstruction, but it argues that outcome reporting in RI-RVF should be multidimensional and that preoperative counselling should be framed accordingly.

Regenerative approaches target the damaged microenvironment rather than the defect alone, drawing on the paracrine and angiogenic properties of adipose-derived cells (57–60). Clinical experience in radiation-induced fistula is nonetheless confined to two patients reported in a single case report, and questions of procedural standardisation, regulatory status, long-term safety and the theoretical risk of pro-angiogenic stimulation in cancer survivors remain unresolved (58–60). SVF should at present be regarded as investigational.

Several limitations of the available literature constrain the conclusions of this review. Because RI-RVF is uncommon, the evidence base consists almost entirely of retrospective series and case reports with small denominators, and no randomised comparison of reconstructive techniques exists. Reported series differ in surgical technique, timing of intervention, diversion practice and aftercare, which precludes direct comparison of success rates. Another major knowledge gap is the lack of information on long-term functional outcomes. While most studies focus mainly on whether the fistula physically closed, they often do not properly evaluate patients’ long-term quality of life, including sexual function, bowel control, and mental well-being. Instruments applied across the reviewed literature are heterogeneous and none is disease-specific for RVF – SF-12, FIQL, IBS-QOL, FSFI and the LARS score have all been used – which precludes pooling and makes cross-study comparison of functional outcome unreliable (65–67). Finally, new regenerative therapies like SVF still lack standardized cell isolation methods, clear dosing guidelines, and long-term safety data. Additionally, prospective clinical studies are needed before these methods can be routinely used in everyday clinical practice. The absence of comparative data also constrains the treatment framework proposed here, which should be read as a structured summary of reported practice rather than as a validated decision algorithm.

This review also has methodological limitations of its own. The primary search was restricted to PubMed and did not include Embase, Scopus or the Cochrane Library; the supplementary Google Scholar search was not systematic. Screening decisions were not documented prospectively at the level of individual records, so the selection process cannot be fully reconstructed, and although eight reviewers took part, records were not screened in duplicate. Eligibility was restricted by language and, for the primary search, by publication date. No review protocol was registered, PRISMA reporting was not applied, no formal risk-of-bias instrument was used, and no formal system was applied to grade the strength of evidence or of recommendations; methodological quality was appraised narratively. Accordingly, this article is presented as a narrative review with a structured literature search, and not as a systematic review or a fully reproducible evidence synthesis. The included evidence is dominated by retrospective series and case reports, with substantial heterogeneity in fistula aetiology, surgical technique, diversion practice and outcome definition, and a considerable proportion – particularly that supporting delayed coloanal anastomosis – is extrapolated from low rectal cancer surgery. Selection and publication bias are therefore likely; publication bias has been formally demonstrated in at least one of the meta-analyses on which we draw (34). These are limitations of the conduct of the review rather than of the underlying literature.

## 5 Conclusion

Radiation-induced rectovaginal fistulas represent one of the most formidable reconstructive challenges in pelvic surgery, for which no universal standard of care exists. The condition is a late consequence of radiotherapy delivered predominantly for cervical cancer, and its risk is determined by cumulative dose to the anterior rectal wall, prior pelvic surgery and re-irradiation – variables that are well characterised in the radiotherapeutic literature but almost never reported in the surgical series on which reconstructive practice rests. Management must be individualised and staged, beginning with exclusion of persistent or recurrent malignancy, and planning should be multidisciplinary, involving colorectal surgery, gynaecologic oncology, radiation oncology and stoma care.

Faecal diversion controls symptoms and prepares the tissue bed but rarely achieves definitive closure on its own. Where reconstruction is feasible, the Martius flap, gracilis interposition and delayed coloanal anastomosis all interpose healthy, non-irradiated tissue into a hypoperfused field, and the choice between them rests on fistula height, defect size, local tissue quality and previous repairs rather than on comparative evidence. Reported success rates should be interpreted conservatively: they derive largely from retrospective series with small denominators, mixed aetiologies, variable diversion practice and differing definitions of success. Gracilis interposition in particular retains value as a salvage option when local tissues are heavily compromised, with outcome depending on patient selection, temporary diversion and prevention of perineal infection. Adjunctive regenerative approaches such as autologous stromal vascular fraction are investigational and may warrant evaluation in carefully designed prospective studies; however, the current evidence is insufficient to support routine clinical use.

Future work should redefine what counts as success. Closure of the tract is necessary but insufficient; stoma-free survival, continence, sexual function, pelvic pain and patient-reported quality of life determine whether a technically successful repair translates into a meaningful outcome for the patient.
