# Supplementary material for: Rectovaginal fistulas after radiation therapy: treatment options and outcomes – a narrative review
Source: Front Oncol. 2026 Sep 7;16:1928527. doi: 10.3389/fonc.2026.1928527 (PMC13591310; doi:10.3389/fonc.2026.1928527)
Supplement: Supplementary file 1 [file DataSheet1.pdf]

Supplementary Material

Rectovaginal fistulas after radiation therapy: treatment options and outcomes – a narrative review

Lachowska K, Jankowska N, Januszek A, Orzelska J, Gierulska N, Kwiatkowska A, Kuchno P, Ozimek D, Kułak K, Tarkowski R

Supplementary Table 1. Studies are grouped according to whether they evaluated radiation-induced rectovaginal fistula specifically, mixed rectovaginal fistula populations, other complex anorectal or perineal fistulas, or outcomes extrapolated from low rectal cancer surgery. Assignment to a group depends solely on the population studied, not on the proportion irradiated: group A comprises studies in which the entire cohort had radiation-induced fistula or radiation injury. Whether the original authors analysed and reported the irradiated patients as a distinct subgroup is recorded separately, as a flag in the RI-RVF column, so that the two properties are not conflated. NR entries reflect incomplete reporting in the source publications rather than omission by the present authors. References not listed in Table 1 were used for anatomical, methodological or background context.

CI, confidence interval; DCAA, delayed coloanal anastomosis; EUA, examination under anaesthesia; FIQL, Fecal Incontinence Quality of Life scale; FSFI, Female Sexual Function Index; ICAA, immediate coloanal anastomosis; IPAA, ileo-anal pouch anastomosis; LARS, low anterior resection syndrome; NR, not reported; QoL, quality of life; RI-RVF, radiation-induced rectovaginal fistula; RT, radiotherapy; RVF, rectovaginal fistula; SF-12, Short Form-12 Health Survey; SVF, stromal vascular fraction. Numbers in parentheses are the reference numbers of the article.

Supplementary Table 1: Principal clinical studies included in this review, grouped by the population studied.

Study, year	Design	Total n	RI-RVF n (%)	Fistula characteristics	Intervention	Diversion	Definition of success	Follow-up	Closure rate	Stoma reversal	Complications	Functional / QoL outcomes	Major limitations
**A. Studies specifically in radiation-induced rectovaginal fistula**													
Zelga 2017 (6)	Single-centre, prospectively maintained database, 2003–2013	50	50 (100);subgroup reported separately: n/a	Median age 60 y (range 40–84); cervical cancer 37 (74%), endometrial 13 (26%); 27 (54%) prior Wertheim–Meigs panhysterectomy; median EBRT 44 Gy (range 31.4–52); cumulative dose to point A ≈84 Gy; median latency from RT 20 months (range 5–240); 13 low, 33 medium, 11 high; diameter <2.5 cm in 48	Faecal diversion alone in 48 (96%), low anterior resection in 2	Yes, by design: loop transversostomy 24 (48%), loop sigmoidostomy 14 (28%), loop ileostomy 9 (18%), end ileostomy 1 (2%)	Absence of vaginal passage of faeces, flatus or mucus and absence of a visible fistula tract on CT with rectal contrast	Median 2 years in outpatient clinic; total observation 3 years (range 1–15)	6/50 (12%) spontaneous	1/6; in the remaining 5 the stoma was not reversed because of perioperative risk or patient refusal	Overall 43% (25/50); reoperation in 15%; one in-hospital death; commonest early complication wound infection, commonest late complication stomal prolapse	Acceptance of Illness Scale median 27 (range 11–39); healed vs persistent 35 vs 27, p = 0.081 (NS). Satisfaction with Life Scale median 22 (range 15–32); 22 vs 21, p = 0.571 (NS); no patient classified as dissatisfied. On multivariable analysis only distance from the anal verge predicted healing (p = 0.031; OR 2.35; 95% CI 1.04–5.29)	Retrospective; diversion alone in 96%, so reconstructive outcomes not assessed; single centre
Liu 2025 (5)	Single-centre retrospective comparative study, 2014–2023	66	66 (100)subgroup reported separately: n/a	Mean age 60.4 ± 11.2 (lap) vs 63.6 ± 9.1 (open); primary disease cervical 27, endometrial 13, rectal 26; location high 40, midzone 26; size small 17, medium 47, large 2; sphincter involvement 19	Diverting colostomy alone, laparoscopic vs open; no fistula repair performed in any patient	Yes, by design; sigmoid 39, transverse 27	n/a — fistula closure was not assessed; no postoperative contrast enema or CT performed	Minimum 6 months (inclusion criterion); authors note this as a limitation	n/a	nr	Surgical-site infection 0/28 lap vs 5/38 open (p = 0.04); reoperation 2 vs 3 (NS); no significant difference in stoma-related complications; no in-hospital mortality	Postoperative stay 6 ± 2.43 vs 8 ± 3.33 days (p = 0.0165); pain score at 24 h 3 (2–5) vs 5 (4–8), p < 0.01; days on analgesics 1.5 ± 0.58 vs 2.8 ± 0.86, p < 0.01; incision length 5 vs 13 cm (p < 0.01)	Fistula closure not an endpoint; no postoperative imaging; minimum follow-up 6 months; non-randomised allocation
Karakayali 2016 (49)	Prospectively collected data, retrospectively analysed; single centre, Dec 2010 – Sep 2013	21	21 (100);subgroup reported separately: n/a	All treated for locally advanced cervical carcinoma with radical hysterectomy plus pelvic/para-aortic lymphadenectomy followed by adjuvant chemotherapy and external-beam radiotherapy; no tumour recurrence; mean interval from end of radiotherapy 11 months; 16 presented with stool per vagina, 5 referred with a diverting colostomy after failed local repair (all 5 had rectal advancement flap, 3 also failed transvaginal repair)	Ultralow anterior resection (n = 11) or abdominoperineal pull-through resection with straight coloanal anastomosis (n = 10)	Protective ileostomy in all; outcomes assessed 6 months after ileostomy closure	Absence of recurrent rectovaginal fistula	Mean 20 months after stoma reversal	No recurrence in any patient (21/21)	All patients underwent ileostomy closure	NR	FIQL depression, lifestyle and embarrassment scores significantly improved; pre- vs postoperative incontinence scores not significantly different; bowel function questionnaire and FIQL applied before surgery and 6 months after ileostomy closure	Small single-centre series; no comparator; short follow-up after stoma reversal
Zhong 2017 (2)	Single-centre retrospective comparative study, Jan 2012 – Aug 2015 (31 screened, 26 analysed)	26 (10 resection, 16 colostomy only)	26 (100);subgroup reported separately: n/a	Cervical cancer 76.9%; EBRT 40–60 Gy in 20–30 fractions plus intracavitary 24–54 Gy in 4–9 fractions; median latency 14.5 vs 12 months; median distance from anus 4.0 cm both groups; median diameter 1.2 vs 1.5 cm; groups comparable except ASA (better in resection group, p = 0.015)	Restorative resection with pull-through coloanal anastomosis and prophylactic colostomy (n = 10) vs simple colostomy (n = 16)	Yes in both arms, by design	Complete remission = RPSAS score of 1 point (symptom-free); continence by Wexner score after reversal	Scheduled at 3, 6, 12, 24 and 36 months	Not the primary endpoint; no spontaneous healing in any of the 16 colostomy-only patients	3/10 (30%) in the resection group; 0/16 in the colostomy-only group; reversals at 9 and 35 months; Wexner scores after reversal 4, 0 and 0	No deaths in the resection group; asymptomatic anastomotic leak on routine colonoscopy in 3/10 (30%), managed conservatively; 6/10 (60%) had delayed reversal or chose a permanent stoma; tumour recurrence in 3/16 (18.8%) of the colostomy group	Symptom scores improved in both groups from 3 months, sustained to 2 years. Resection superior for complete remission of tenesmus (6 mo 33.3% vs 0%, p < 0.05; 12 mo 66.7% vs 16.7%, p < 0.05) and perineal mucous discharge (6 mo 88.9% vs 6.7%, p < 0.001; 12 mo 77.8% vs 15.4%, p < 0.01; 24 mo 85.7% vs 25.0%, p < 0.05)	n = 26; non-randomised, groups differed in ASA; single centre
Kim 2021 (60)	Case report (2 cases), single centre	2	2 (100;subgroup reported separately: n/a)	Both after chemoradiotherapy for rectal cancer, not gynaecological malignancy. Case 1: 61 y, 5000 cGy, robot-assisted low anterior resection, RVF after ileostomy reversal, one failed repair attempt. Case 2: 59 y, short-course 2500 cGy, laparoscopic low anterior resection with hysterectomy and BSO, RVF at 3 months, one failed primary repair	Case 1: debridement, vaginal mucosal Limberg flap, 3 mL SVF injected into posterior vaginal wall. Case 2: two staged procedures — 5 mL SVF with 30 mL fat graft, then 28 mL cell-assisted lipotransfer with 5 mL SVF three months later	Case 1: loop ileostomy created at the time of surgery, later reversed. Case 2: none	Case 1: no air leak or discharge on rectoscopy, no clinical recurrence. Case 2: no clinical recurrence and no residual fistula on MRI	Case 1: 12 months. Case 2: 5 months after the second procedure	2/2	1/1 (case 1) case 2 had no stoma	NR	NR	Two cases; both after rectal rather than gynaecological cancer; short follow-up in one

continued on next page

Table 1 (continued)

Study, year	Design	Total n	RI-RVF n (%)	Fistula characteristics	Intervention	Diversion	Definition of success	Follow-up	Closure rate	Stoma reversal	Complications	Functional / QoL outcomes	Major limitations
Smirnov 2024 (13)	Case report	1	0 - chronic post-radiation proctitis, not fistula;subgroup reported separately: n/a	n/a. 76-year-old man treated for prostate adenocarcinoma cT3N0M0 with two courses of proton therapy, total equivalent dose 90.6 Gy; erosive-ulcerative proctitis RTOG–EORTC grade 4 with recurrent profuse bleeding; multiple posterior-wall ulcers 7–14 mm; five months of conventional therapy had failed	Endoscopic submucosal and pararectal injection of enzymatically isolated autologous SVF (300 mL lipoaspirate; SVF containing ≥10% MSC by CD44/CD90)	None	Endoscopic and histological resolution of ulceration; absence of bleeding	12 months	n/a — complete epithelialisation of ulcers at 1 month; proctitis remission with neoangiogenesis at 6 months	n/a	None; uneventful course, discharged day 3	No bleeding episodes over 12 months; no laboratory evidence of chronic anaemia	Single case; radiation proctitis rather than fistula; male patient after prostate irradiation
**B. Mixed-aetiology rectovaginal fistula populations**													
Corté 2015 (41)	Prospective institutional database, retrospectively analysed; single centre, 1996–2014	79 patients, 286 procedures (mean 3.6 ± 2.5 per patient, range 1–11)s	4 (5%) — radiation proctitis. Crohn 34 (43%), postoperative anastomotic leakage 25 (32%), obstetric 7 (9%), unknown 4 (5%), pelvic cancer 2 (3%), diverticulitis 2 (3%), trauma 1 (1%);subgroup reported separately: no	Mean age at diagnosis 43 ± 16 y; 55 (70%) low fistulas; 26 (33%) referred after failed repair elsewhere; mean interval diagnosis to first repair 22 ± 45 months	217 conservative (76%): seton 59, vaginal flap 49, rectal flap 46, stoma alone 27, plug 15, glue 13, other 8. 69 major (24%): gracilis 32, biomesh 9, delayed coloanal with pull-through 11, standard coloanal/ colorectal 8, abdominoperineal excision 9	67 patients (85%) had a stoma at some point; 152/286 procedures (53%) performed in a diverted patient	Absence of any vaginal passage of faeces, flatus or mucus for at least 3 months after the last procedure and absence of stoma. Abdominoperineal excision and permanent stoma counted as failure	Median 33.1 months (range 4–190)	Per patient 57/79 (72%); per procedure 57/286 (20%). Cumulative 48% at 1 year, 57% at 2 years. Per procedure: seton 5%, glue/plug 11%, advancement flaps 12%, stoma alone 15%, gracilis 50%, biomesh 44%, standard anastomosis 50%, delayed coloanal with pull-through 91% (10/11)	Continuity restored in 50/67 (75%); permanent stoma in 17/79 (22%)	NR as a separate endpoint	Wexner assessed in 33/57 successes; mean 2.7 ± 2.9; 22 with no or minimal incontinence (<5), 9 moderate (5–9), none severe (≥10). Multivariable predictors of success: major procedure OR 6.4 (2.9–14.2, p < 0.001), diverting stoma OR 3.5 (1.4–8.7, p = 0.009), <9 months to first surgery OR 2.3 (1.1–5.3, p = 0.046), primary management in-house OR 3.2 (1.5–6.9, p = 0.003)	Only 4/79 (5%) radiation-induced; aetiology not an independent predictor, but underpowered for this subgroup; retrospective, single centre
Frontali 2021 (15)	Bicentric retrospective study on a prospective database; Bologna and Clichy, 2003–2019	61 (51 RVF, 10 pouch-vaginal fistula after IPAA);	3 (5%) with previous radiotherapy; cancer as aetiology in 4 (7%);subgroup reported separately: yes	Mean age 42 ± 12 y (24–72); Crohn 30 (49%), ulcerative colitis 8 (13%), obstetric 10 (16%), trauma 9 (15%), cancer 4 (7%); mean interval diagnosis to graciloplasty 59 ± 59 months; 57/61 (93%) had ≥1 prior repair attempt, mean 2.4 ± 1.7	First graciloplasty; second procedures excluded	100% — all patients had a loop ileostomy before or at graciloplasty; 30 (49%) at the time of the procedure, 31 (51%) already in place	Failure = any vaginal discharge of faeces, flatus or mucus and/or absence of stoma closure	Mean 56 ± 48 months (range 1–183)	Failure 24/61 (39%); by aetiology: Crohn 43% (13/30), pouch-vaginal 38% (3/8), obstetric 30% (3/10), post-radiotherapy 33% (1/3), other 40% (4/10) — not significant	Implicit in the definition of failure	Clavien–Dindo ≥II in 21/61 (34%); grade II 18/61 (30%), grade IIIb 3/61 (5%); no grade IV or V; perineal complication 15/61 (25%); median stay 8 ± 7 days	Not assessed. Univariable risk factors for failure: absence of postoperative antibiotic prophylaxis (3/24 vs 18/37, p = 0.0053; failure 14% with prophylaxis vs 53% without, p = 0.0058) and postoperative perineal infection (11/23 vs 4/37, p = 0.0021)	Only 3/61 (5%) post-radiotherapy; univariable analysis only; all patients diverted, so effect of diversion could not be assessed
Söderqvist 2022 (65)	Observational study, retrospective records with prospective HRQoL follow-up; Uppsala, 2003–2018; registered NCT05006586	55;	1 (1.8%) — radiation; plus 2 of unknown aetiology in the same "others" category;subgroup reported separately: yes	Median age 40 y (IQR 33–49), mean BMI 27; obstetric 18 (32.7%), cryptoglandular infection 17 (30.9%), Crohn 7, anastomotic 4, iatrogenic 6, unknown 2, radiation 1; low 27 (49.1%), intermediate 17, high 3, uncategorised 8; 24 (43.6%) had ≥1 previous repair; 41/55 referred from other hospitals	Index procedure: ERAF 24 (43.6%), transperineal ELC 9, rectal ELC 6, sphincteroplasty 5, LIFT 2, gracilis 1, other 8. Across all 122 attempts: local repair 51, ELC 40, gracilis 10, other 21	Stoma at index surgery in 15 (27.3%); 12 more later; no effect on healing — index 21.4% vs 29.3% (p = 0.734), final 53.3% vs 72.5% (p = 0.185)	Absence of signs and symptoms of persistent or recurrent fistula at last follow-up, by surgeon's assessment	Median 30.2 months (IQR 12.5–51.7)	Index 14/55 (25.5%); final 37/55 (67.3%). By aetiology — traumatic 45.9% index / 91.7% final; inflammatory 7.1% / 46.4%; others 33.3% / 66.7% (p = 0.004 and p = 0.001). The single radiation patient did not heal (0/1). Per attempt: gracilis 5/10 (50%), local repair 33.3%, ELC 30%, other 4.8% (p = 0.019)	9 patients never had reversal; 4 received a permanent stoma because of the fistula	15/55 (27.3%) after index surgery; Clavien–Dindo I 8 (14.5%), II 7 (12.7%), IIIb 1 (1.8%)	RAND-36 in 33/55. Unhealed scored lower than healed and than the Swedish norm in 6/8 domains, not significant. PCS 70.6 ± 25.0 vs 64.7 ± 36.1 (p = 0.85); MCS 74.2 ± 18.3 vs 66.1 ± 17.8 (p = 0.28). Whole cohort scored below the general population in every domain. Pelvic pain 18/55; vaginal flatulence 24/55; abnormal discharge 34; stool per vagina 33; recurrent UTI 9	Only 1/55 radiation-induced, and that fistula did not heal; HRQoL assessed in 33/55; single centre
Muller 2023 (43)	Retrospective analysis of a prospectively maintained database; Beaujon Hospital, Clichy, 2005–2021	19 patients, 45 procedures	1 (5%) - neoadjuvant irradiation for rectal cancer. Crohn 10 (53%), iatrogenic 5, post-partum 3, idiopathic 1;subgroup reported separately: no	Median age at graciloplasty 39 y (25–64); 5 (26%) active smokers; mean interval graciloplasty to recurrence 3 ± 3 months; all had a stoma at the time of graciloplasty, only 3 (16%) closed before recurrence	Salvage after failed graciloplasty: transanal repair 31 (69%), DCAA 4 (9%), biological mesh 3, second graciloplasty 3, stoma alone 2, redo IPAA 2; one patient managed medically	Covering stoma in all procedures	Absence of fistula (no vaginal discharge of faeces, gas or mucus) and absence of stoma at last follow-up	Mean 59 ± 44 months after salvage surgery; 66 ± 50 months from gracilo-plasty	11/18 (58%). By procedure: DCAA 4/4 (100%), local repair 5/31 (16%), stoma alone 1/2 (50%), redo IPAA 1/2 (50%). Second graciloplasty 0/3 and biological mesh 0/3 both failed entirely	Implicit in the success definition; 8 patients ended with a permanent stoma and/ or active fistula	3/19 (16%), all Clavien–Dindo II: two abscesses after local repair, one rectocutaneous fistula after rectal flap; no mortality	Among the 4 DCAA patients: no recurrence; 2 reported occasional flatus incontinence and rare faecal incontinence, one normalised after sacral neuromodulation. Risk factors for failure: Crohn disease 2/10 (20%) vs 9/9 (100%) other aetiologies (p = 0.02); minor repair 6/39 (15%) vs abdominal surgery 5/6 (83%) (p = 0.002)	n = 19; only 1/19 radiation-related; salvage cohort only, not applicable to first-line repair

continued on next page

Table 1 (continued)

Study, year	Design	Total n	RI-RVF n (%)	Fistula characteristics	Intervention	Diversion	Definition of success	Follow-up	Closure rate	Stoma reversal	Complications	Functional / QoL outcomes	Major limitations
Trompetto 2019 (27)	Prospectively recorded, retrospectively analysed; single surgeon, single centre, 2009–2017	24 (from 97 women undergoing 195 procedures in the same period)	2 (8.3%) — after anterior rectal resection plus pelvic radiotherapy;subgroup reported separately: no	Mean age 46.1 ± 13.0 y; BMI 25.5 ± 5.7; smokers 7 (29.2%); postpartum 10 (41.7%), post-bartholinitis 4 (16.7%), IBD 6 (25%), cryptoglandular 1, iatrogenic in AIDS 1; all had ≥2 previous repairs (mean 2.75 ± 1.82); diameter 2.25 ± 0.62 cm (1.0–3.5); minimum distance from introitus 2.4 ± 0.59 cm	Martius flap (bulbocavernosus interposition), all by the same surgeon	100% — before (15, 62.5%) or during (9, 37.5%) the procedure. Authors consider end-colostomy mandatory	Absence of subjective symptoms and of the fistula, confirmed by examination under anaesthesia	Mean 42 ± 29 months (3–101); 19 (79%) beyond 24 months	22/24 (91.6%); 2 failures, 2 recurrences at 18 and 24 months	22/24, at a median of 5.5 months (IQR 3.0–7.5); one Crohn patient chose to retain the stoma despite healing	1/24 (4.2%) — paravaginal wound dehiscence requiring re-suturing under local anaesthesia; no major complications; median stay 3.5 days	No episodes of dyspareunia. CCIS unchanged (1.0, range 0–3.5). SF-12 physical 33.6 ± 7.2 → 50.8 ± 7.8 ($p < 0.001$); mental 32.6 ± 6.7 → 56.3 ± 7.8 ($p < 0.001$). FSFI 19.5 ± 6.6 → 24.4 ± 6.3 ($p < 0.001$). Mental component improved even in patients whose repair failed or recurred, while the physical component did not	Only 2/24 radiation-induced; single surgeon; all patients had ≥2 previous repairs, limiting generalisability
Collard (GREC-CAR) 2025 (55)	Multicentre retrospective cohort 16 GRECCAR centres, 2010–2023	78	8 (10) as the cause of fistula; 38 (49) with any history of pelvic radiotherapy;subgroup reported separately: yes	Median age 54.5 y (IQR 42.5–64.8); 97% ASA 1–2; causes: chronic leakage of colorectal/coloanal anastomosis 52 (67%), surgical injury 14 (18%), radiotherapy 8 (10%), Crohn 4 (5.1%); vaginal opening lower third 30, middle 25, upper 23; median diameter 1.0 cm (IQR 1.0–2.0); 31 (40%) had ≥1 failed prior treatment	Delayed coloanal anastomosis; median interval between stages 11 days (IQR 7–16); laparotomy 63%, laparoscopic 27%, robotic 10%	Present before DCAA in 63 (81%); additional diverting stoma at DCAA in 51 (65%)	No 30-day mortality, no fistula recurrence at latest follow-up, and preserved bowel continuity	Median 31 months	63/78 (81%); 92% (60/65) without anastomotic leakage vs 23% (3/13) with leakage ($p < 0.001$)	Implicit in the success definition; 9 patients retained a permanent stoma because of leakage, 3 because of poor function	Overall 45%; major (Clavien-Dindo ≥III) 23%; anastomotic leakage 13/77 (17%); intra-abdominal colon necrosis 2.6%; one postoperative death; median stay 16 days	LARS assessed in 57/67 eligible; median LARS 25.0/42 (IQR 15.2–35.0); good function (no/minor LARS) 33/54 (58%); 42% (24/57) had major LARS or a stoma for poor function; quality of life and sexual function not directly assessed	Mixed-aetiology cohort; only 8/78 (10%) radiation-induced, and of these 4 had been treated for anal canal cancer, 3 for cervical and 1 for rectal cancer; success by radiotherapy history reported only on univariate analysis, without multivariable adjustment; retrospective
Ryoo 2019 (8)	Prospectively registered, retrospectively reviewed; single tertiary centre (Seoul National University), 1998–2016	92 patients, 128 operations;	27 (29.3%) — the single most common cause. Radiotherapy for cervical cancer 20 (74.1%), rectal cancer 6 (22.2%), bladder cancer 1 (3.7%). A further 26 (28.3%) after pelvic operation;subgroup reported separately: yes	Median age 49 y (16–80); low fistula 58 (63.0%), high 34 (37.0%); median interval from radiotherapy to fistula 11 months (range 0–336); high fistulas significantly associated with radiotherapy ($p < 0.001$) and cancer invasion ($p = 0.002$); stool per vagina in 83 (90.2%)	First operation: diverting ostomy alone 53 (57.6%), transanal rectal advancement flap 25 (27.2%), sphincteroplasty with perineoplasty 5, fistulectomy with seton 4, bowel resection 4, Martius flap. Repeat operations in 16 (17.4%) including graciloplasty, Martius flap, stem cell injection	Ostomy alone as first procedure in 53 (57.6%); protective ostomy in 12 oof 16 patients	Closure confirmed by symptoms, digital rectal examination or fistulogram at follow-up	Median 17 months (range 0–123)	After first operation 31/92 (33.7%); overall 39/92 (42.4%); after repeat surgery 8/16 (50%). In the radiotherapy group only 2 of 27 patients achieved closure, both with low fistulas — one after advancement flap, one after graciloplasty	45/92 (48.9%) never had their diverting ostomy taken down. Among repeat-operation patients, take-down in 6/16 (37.5%). No patient treated with diverting ostomy alone had the ostomy reversed during follow-up	NR	Not assessed. Multivariable analysis: radiotherapy ($p = 0.010$) and pelvic operation ($p = 0.045$) were independent poor prognostic factors; Crohn disease showed a trend ($p = 0.058$)	Retrospective; heterogeneous interventions across the cohort; closure achieved in only 2/27 irradiated patients, both with low fistulas, limiting inference for high fistulas
**C. Other complex anorectal or perineal fistulas (indirect evidence)**													
Swindon 2024 (26)	Systematic review and proportional meta-analysis; 12 non-randomised studies (11 retrospective, 1 prospective)	137 Martius flap procedures (44 primary, 93 recurrent); mean age 42.4 ± 15.7 years;	26 procedures from 2 series (1982, 1988), both pre-1990;subgroup reported separately: yes	All low RVF; only 3 of 12 studies specified both location and size	Martius flap	Variable; 4 of 12 studies used no stoma	Complete fistula healing (definitions varied across included studies)	Range 3–128 months across studies; not pooled	Primary 91.4% (95% CI 79.5–98.5); recurrent 77.5% (95% CI 62.2–89.7); radiation subgroup 94.6% (95% CI 83.3–99.8; $I^2 = 0\%$)	NR	Complications 29% (95% CI 9.0–54.7; $I^2 = 85.4$); recurrence 12.0% (95% CI 5.0–21.9)	Dyspareunia 6/137 (4.5%); only 2 of 12 studies used objective measures of sexual function	Radiation subgroup derived from 26 procedures in two series published in 1982 and 1988; high heterogeneity (I^2 85% for complications); 7/12 studies at serious risk of bias on ROBINS-I

continued on next page

Table 1 (continued)

Study, year	Design	Total n	RI-RVF n (%)	Fistula characteristics	Intervention	Diversion	Definition of success	Follow-up	Closure rate	Stoma reversal	Complications	Functional / QoL outcomes	Major limitations
Garoufalia 2023 (34)	Systematic review and proportional meta-analysis; PROSPERO CRD42022 302311; 25 studies published 2002–2021 (23 retrospective, 2 prospective non-randomised)	658 (426 women);	119 (~18%) with a history of radiotherapy. Fistula types: rectovaginal 50.7%, rectourethral 33.7%. Causes: pelvic surgery 29.4%, IBD 25.2%;subgroup reported separately: yes	503/563 (89.3%) had previous repairs; median number 1–8; 75.3% had at least one prior repair	Gracilis muscle interposition for complex perineal fistula	Protective stoma in 13 studies; stoma was not significantly associated with success on meta-regression	Not clearly defined across studies; in most, absence of discharge of faeces, flatus or mucus through the fistula without a diverting stoma. Overall success defined as final rate including additional procedures	Median 4 months to 4.6 years; median stay 6.5–23 days	Crude 498/658 (75.7%); weighted 79.4% (95% CI 73.8–85.0, I^2 = 75.3%). Index procedure 62.8% (95% CI 40.5–85.2); after additional procedures 84.5% (95% CI 74.1–95.0). RVF subgroup 74.9% (95% CI 67.0–82.9); radiation subgroup 83.9% (95% CI 70.4–95.5); follow-up <2 y 82.3%, ≥2 y 75.1%	Implicit in the success definition	Short-term 25.7% (95% CI 18.1–33.2); long-term 3% (95% CI 0.8–5.2); 30-day reoperation 3.6% (95% CI 1.6–5.6); recurrence 16.7% (95% CI 11.0–22.3). Two deaths, unrelated to the procedure	Not assessed. Meta-regression: female sex (p = 0.001), rectovaginal fistula (p = 0.003), pouch fistula (p = 0.03) and IBD (p = 0.04) predicted failure	Only ~18% with radiotherapy history; heterogeneous fistula types including rectourethral; certainty of evidence graded very low; significant publication bias (Egger p = 0.001)
Schoene 2023 (16)	Retrospective, prospective database + telephone interview; single centre, 2005–2021	60 (44 women, 16 men), 63 proce-dures;	17 (28.3) irradiation; abscess 14, obstetric 8, iatrogenic/ unknown 21; IBD excluded;subgroup reported separately: yes	Mean age 50 y (24–82); 59/60 (98%) had previous failed repairs, mean 3.6 (1–15); 35 of 61 fistulas were rectovaginal	Gracilis muscle transposition (Baeten tunnel technique)	100% — stoma in all patients before or during surgery	Primary healing: no recurrence within 3 months, confirmed clinically and endoscopically	Mean 35.9 months (1–187); 43.4 months in the irradiated group	Overall primary 20/60 (33%), final 39/60 (65%). Irradiated subgroup: primary 6/17 (35.3%), final 8/17 (47.1%) — lowest of four groups (obstetric 87.5%, iatrogenic 76.2%, abscess 57.1%)	36/60 (60%); 7/17 in the irradiated group	15/60 (25%): wound healing disorder 6, gracilis necrosis 3, incisional hernia 2, scar pain 2, suture granuloma 1, osteomyelitis 1	Not assessed. Early recurrence in irradiated subgroup 7/17 (41.2%), late 4/17 (23.5%))	Retrospective; IBD excluded; 98% had failed previous repairs, limiting generalisability to first-line repair
Oner 2025 (35)	Retrospective single-institution review, all operations by one colorectal surgeon, 10-year period	18 patients, 19 flaps	13/18 (72.2) had prior radiotherapy (external beam 10, brachytherapy 3), but only 7 fistulas were rectovaginal; primary malignancy was prostate 7, rectal 3, anal 3, bladder 1subgroup reported separately: no	Median age 60 y (25–72); 8 men, 10 women; mean BMI 28; indications: complex fistula 14 (77.8%) — rectovaginal 7, rectourethral 6, urethrocutaneous 1 — and perineal wound coverage 4 (22.2%); 6/14 (42.9%) had failed prior repair	Gracilis flap harvested and interposed by the colorectal surgeon, without plastic surgery involvement	100% — stoma pre-existing or placed at operation; temporary in 12 (66.7%), permanent in 6 (33.3%)	Flap success, i.e. absence of persistent fistula or wound failure	Median 24 months (1–81)	Primary flap success 15/18 (83.3%); flap failure 3 (16.7%) — 2 rectourethral, 1 rectovaginal; both rectourethral healed after further intervention	11/12 temporary stomas closed (91.7%); one converted to permanent colostomy	3/18 (16.7%): deep venous thrombosis 2, wound infection 1; readmission 2 (11%); median stay 5 days (3–20)	Not assessed	n = 18, mixed sex; only 7 rectovaginal fistulas; proportion of these that were radiation-induced not reported
Kersting 2019 (39)	Retrospective records review with postoperative questionnaire and clinical examination; single centre, 2011–2016	19;	1 (5.3) radiogenic; postoperative 8 (42.1), Crohn 6 (31.5), idiopathic 4 (21.1);subgroup reported separately: yes	Mean age 48 y (20–79); all recurrent; mean 4 previous operations (1–15), 82% performed elsewhere; 15/19 had a stoma preoperatively; mean disease duration 2.5 y	Gracilis muscle transposition, used only as last resort after failed prior repair	100% — protective stoma in all patients post-operatively	Fistula closure confirmed clinically and by endoscopic ultrasound	Mean 23 months (7 months–3.5 years)	Primary 10/19 (53%); final 14/19 (74%). The single radiogenic fistula did not heal (0/1). By aetiology: idiopathic 4/4 (100%), postoperative 7/8 (88%), Crohn 3/6 (50%). By previous operations: 1 prior 83%, 2 prior 100%, ≥3 prior 60%	11/19 at time of survey; interval 3–19 months (mean 8)	5/19 (26%): perineal wound healing disturbance 3, thigh wound disturbance with anal abscess 1, phlegmonous thigh wound with perineal abscess and recurrence 1; no intraoperative complications; mean stay 11 days	Sexual function deteriorated in 15/19 (79%), unchanged in 3, improved in 1; only 4 were sexually active preoperatively, 2 postoperatively; pain was the main reason for cessation both before (57%) and after (53%). Wexner 11.8 preop (n = 4) vs 9.2 postop (n = 11). Quality of life improved in 11/19 (58%), worsened in 5, unchanged in 3; 15/19 (79%) would undergo the operation again. Donor-site morbidity in 8/19 (42%): sensory disturbance 8, movement problems 5, thigh pain 4; unsightly scarring 14/19 (74%)	n = 19; only 1 radiogenic fistula, which did not heal; sexual function assessed in a small subgroup

continued on next page

Table 1 (continued)

Study, year	Design	Total n	RI-RVF n (%)	Fistula characteristics	Intervention	Diversion	Definition of success	Follow-up	Closure rate	Stoma reversal	Complications	Functional / QoL outcomes	Major limitations
Grott 2021 (42)	Follow-up study, partly prospective database, partly retrospective; single tertiary referral centre, Feb 2006 – Jan 2016	46 patients, 47 gracilis transpositions	Malignant underlying disease in 19/46 (41%); among 31 questionnaire respondents, 8/31 (26%) with successfully closed fistula had received radiotherapy. Prior radiation was not a negative predictor of healing (p = 0.1214);} subgroup reported separately: no	29 (neo-)rectovaginal, 4 pouch-vaginal, 3 perineal, 3 rectovesical, 3 urethroperineal, 2 urethrorectal, 2 intestinoperineal; mean 2 previous repair attempts (range 2–12); mean age 54.6 y	Gracilis muscle transposition; 45/47 by one surgeon	44/46 (96%); only 2 birth-trauma patients operated without stoma, both healed	Fistula closure verified by rectoscopy and contrast enema ~3 months postoperatively	Mean 73.4 months (median 76, range 26–145)	Primary 25/47 (53%); final 34/46 (74%). Recurrence at mean 276 days (median 180, range 46–1287). Birth trauma had the highest closure rate (p = 0.0045)	14/15 reversible stomas closed (93%) in the successful group; 15/31 respondents still had a stoma, 8 because of persistent fistula	11/47 (23%), including 9 Clavien–Dindo IIIb; 2 muscle necrosis, 1 muscle dislocation; no deaths	Wexner median 10 (range 3–14) — moderate incontinence. FIQL reduced across all four domains. SF-12 only slightly below the German general population; no difference by fistula status, stoma, or underlying disease. FSFI mean 16.25 (SD 10.59), rising to 18.2 in healed patients — well below the 26.55 dysfunction cut-off; FSFI 4.2 with stoma vs 19.5 without (p = 0.03). All successfully treated patients would recommend the operation; none of the unsuccessfully treated would	Retrospective; heterogeneous fistula types; questionnaire completed by 31/46; single surgeon in 45/47 cases
Picciariello 2020 (40)	Retrospective study on a prospectively maintained database; single tertiary colorectal unit, 2003–2017	14 (9 female, 1 transgender, 4 male), selected from 47 operated for RVF/RUF	1 (7.1) — neoadjuvant radiotherapy for gynaecological malignancy; a further patient had RUF after prostate radiotherapy. Cancer-related fistulas were an exclusion criterion;subgroup reported separately: no	Median age 48 y (29–76); iatrogenic 11, Crohn 3; 9 patients had 1–3 previous failed repairs; 3 had preoperative faecal incontinence; high (fornix) and ano-vestibular fistulas excluded	Graciloplasty; gracilis wrapped around the anal canal as neosphincter in patients with severe incontinence	Not scheduled; 4/14 already had a colostomy from previous surgery elsewhere	Complete healing on clinical examination of vagina and anorectum	Median 1 year (IQR 10–14 months)	11/14 (78%); 2/3 Crohn fistulas healed	2/4	No mortality, no major complications; wound infection in 3 patients, managed conservatively; median stay 6 days (IQR 4–9)	SF-36 improved in all domains at 6 months except "body pain". CSFQ rose from 35.5 (IQR 31–38.7) to 44 (IQR 37.7–48.5), p = 0.03, in the 8 patients still sexually active. CPGAS improved from 0 to 4 ("moderately better"). Wexner fell from 12 (IQR 11–14) to 5 (IQR 4–5), p = 0.01, in the 5 with preoperative incontinence	n = 14; cancer-related fistulas excluded by design; median follow-up 1 year
El-Gazzaz 2010 (66)	Prospective database with long-term telephone follow-up; single centre, 1997–2009	100 (of 268 women with RVF identified)	0 — obstetric 60 (60%), cryptoglandular 40 (40%); subgroup reported separately: n/a	Mean age 42.8 ± 10.5 y; BMI 28.8 ± 7.6; low RVF; commonest complaints vaginal drainage 79%, gas per vagina 65%, stool per vagina 52%; faecal incontinence preoperatively in 42, more often with obstetric aetiology (76% vs 24%, p < 0.05)	Various repairs including episioproctotomy, local repair and tissue transposition	Seton or stoma used in a proportion; neither affected healing	Healing, assessed from prospective database and telephone contact	Mean 45.8 ± 39.2 months	68/100 (68%). On multivariable analysis, failure was associated with higher BMI (p = 0.001) and number of previous repairs (p = 0.02); age, smoking, comorbidity, seton and stoma had no effect. Episioproctotomy healed better on univariable but not multivariable analysis	NR	NR	Dyspareunia in 12/47 (25.5%) of sexually active women. Faecal and sexual function and quality of life were comparable between healed and unhealed patients. Instruments: SF-12, FIQL, IBS-QOL, Female Sexual Function Index	No radiation-induced fistulas; obstetric and cryptoglandular aetiology only
**D. Extrapolated from low rectal cancer surgery (indirect evidence)**													
La Raja 2022 (52)	Systematic review and meta-analysis; PROSPERO CRD42021 242938; 9 studies in qualitative synthesis, 4 in meta-analysis; studies 2000–2020	905 (386 DCAA, 519 ICAA)	0 (low rectal cancer only);subgroup reported separately: n/a	n/a. Neoadjuvant chemoradiotherapy or radiotherapy in 249/386 (65%) DCAA and 458/519 (88%) ICAA	Turnbull-Cutait DCAA with pull-through vs ICAA with diverting stoma after TME	ICAA with loop ileostomy by design; DCAA without primary stoma	n/a — endpoints were complications, pelvic sepsis and definitive stoma	Variable across studies	n/a	Definitive stoma 2% vs 2%; OR 0.77 (95% CI 0.15–3.85), p = 0.75, I^2 = 0%	Clavien–Dindo ≥III: 13% DCAA vs 21% ICAA; OR 1.17 (95% CI 0.38–3.62), p = 0.78, I^2 = 20%. Pelvic sepsis 7% vs 14%; OR 0.37 (95% CI 0.16–0.85), p = 0.02, I^2 = 0%	Quantitative synthesis not possible — scores too heterogeneous (Wexner, LARS, Hida, Kirwan, simple stool-frequency scores). No significant difference between groups across studies	Low rectal cancer, not fistula population; only 4 studies in quantitative synthesis; wide confidence intervals

continued on next page

Table 1 (continued)

Study, year	Design	Total n	RI-RVF n (%)	Fistula characteristics	Intervention	Diversion	Definition of success	Follow-up	Closure rate	Stoma reversal	Complications	Functional / QoL outcomes	Major limitations
Wang 2026 (53)	Systematic review and meta-analysis; PROSPERO CRD42025 1233006; 16 studies (2 RCTs, 14 retrospective), search to October 2025	1409 (822 ICAA, 587 DCAA)	0 (low rectal cancer);subgroup reported separately: n/a	n/a; tumour $\leq$7 cm from anal verge	DCAA vs ICAA with prophylactic ileostomy	ICAA with prophylactic ileostomy by design	n/a	Mostly $\leq$3 years	n/a	NR	Total OR 2.74 (95% CI 1.89–3.98, $p < 0.00001$); anastomosis-related OR 3.46 (95% CI 2.32–5.15, $p < 0.00001$); anastomotic leakage OR 2.79 (95% CI 1.71–4.57, $p < 0.0001$); pelvic infection OR 2.56 (95% CI 1.29–5.10, $p < 0.01$) — all favouring DCAA. No difference in operative time, blood loss, length of stay, anastomotic bleeding, stricture, obstruction or urinary retention	Not assessed. No difference in local recurrence (OR 1.02) or distant metastasis (OR 1.51)	Low rectal cancer, not fistula population; 14/16 studies retrospective
Kitaguchi 2025 (54)	International multicentre retrospective cohort; Asian Colorectal Cancer Collaborative	305 (109 DCAA, 196 ICAA))	0 (low rectal cancer);subgroup reported separately: n/a	n/a	Elective minimally invasive TME with hand-sewn coloanal anastomosis, delayed vs immediate	ICAA with diverting stoma; DCAA without stoma	n/a— primary outcome was the overall 30-day complication rate	Functional assess-ment at 1 and 2 years	n/a	n/a — DCAA avoids stoma creation altogether	Overall 30-day 25%; DCAA 15% vs ICAA 31% ($p = 0.002$). Early anastomosis-related 7% vs 15% ($p = 0.047$); late 2% vs 11% ($p = 0.005$)	At 2 years: major LARS 38% vs 60% ($p = 0.018$); severe incontinence 0% vs 8% ($p = 0.029$). Instruments: LARS score and Wexner score	Low rectal cancer, not fistula population; retrospective, non-randomised allocation; DCAA performed without stoma, so functional advantage cannot be attributed to anastomotic technique alone
Zheng 2017 (24)	Single-centre retrospective cohort; Fudan University Shanghai Cancer Center, 2006–2016	1493 women undergoing low anterior resection; 24 developed RVF	0 as a cause. Among the 24 with RVF, 6 (25%) had received neoadjuvant radiotherapy and 2 adjuvant radiotherapy started after the fistula appeared. Neoadjuvant radiotherapy was not a risk factor for RVF ($p = 0.10$);subgroup reported separately: yes	Incidence 24/1493 (1.61%). Onset 3–1161 days postoperatively, median 19.5 days; 79% within 30 days. Mean distance from anal verge 6.0 cm in RVF vs 7.98 cm without ($p < 0.001$)	Supplemental diverting stoma in 21/24 (87.5%): transverse colostomy 18, colostomy plus fistula suture 1, terminal ileostomy 2. One patient had suture alone	Prophylactic stoma in 5/24; prophylactic ostomy was not associated with RVF occurrence	No faeces or gas per vagina, confirmed by examination, negative methylene blue test and absence of fistula on colonoscopy	Median 1774 days (range 228–3733)	16/24 (66.7%) overall; 15/21 (71.4%) after diversion alone. Healing time 30–1225 days (median 210)	3 died before stoma closure; 2 with prophylactic stoma had not undergone closure at the time of writing	n/a — RVF was itself the outcome. Hospitalisation 27.0 vs 18.0 days ($p = 0.005$); cost USD 8115 vs 5982 ($p = 0.003$)	Not formally assessed. Multivariable analysis: unsatisfactory anastomosis HR 6.474 (95% CI 2.236–18.743, $p = 0.001$) and distance from anal verge HR 0.650 (95% CI 0.496–0.851, $p = 0.002$)	Colorectal cancer population; fistula arose as anastomotic complication, mechanistically distinct from radionecrosis

continued on next page

Table 1 (continued)

Study, year	Design	Total n	RI-RVF n (%)	Fistula characteristics	Intervention	Diversion	Definition of success	Follow-up	Closure rate	Stoma reversal	Complications	Functional / QoL outcomes	Major limitations
Ge 2023 (48)	Systematic review, meta-analysis and trial sequential analysis; PROSPERO CRD42022 303133; 30 RCTs reviewed, 5 pooled	317 in the meta-analysis (157 ileostomy, 160 colostomy); 3679 across all 30 RCTs	0 (preventive diversion in colorectal surgery);subgroup reported separately: n/a	n/a	Preventive loop ileostomy vs loop colostomy	Yes, by design	n/a — complication rates were the endpoint	Median time to stoma closure 15–183 days across studies	n/a	n/a	Prolapse 1.27% (2/157) ileostomy vs 15.63% (25/160) colostomy; OR 0.10 (95% CI 0.03–0.34), p = 0.0002 — the only difference confirmed on trial sequential analysis. No significant difference in leak (OR 0.60, 95% CI 0.32–1.15), wound infection (OR 0.60, 95% CI 0.26–1.41) or ileus (OR 1.14, 95% CI 0.44–2.95). In the wider 30-trial comparison, ileostomy was associated with more skin irritation, high output, ileus and pneumonia; colostomy with more prolapse, parastomal fistula and enterocutaneous fistula	n/a	Preventive diversion in colorectal surgery; not a fistula population; only 5 of 30 trials pooled
